# Zhimu-Huangbai codecoction for the treatment of type II diabetes mellitus through its self-assembling nanoparticles

**DOI:** 10.1186/s13020-025-01290-z

**Published:** 2026-01-07

**Authors:** Wenlong Nie, Meifang Jiang, Yin Li, Jinshuai Lan, Zhe Li, Zhijun Bi, Donghao Gu, Minquan Zhang, Yue Ding, Tong Zhang

**Affiliations:** 1 School of Pharmacy, Shanghai University of Traditional Chinese Medicine, Shanghai, 201203 China; 2 SPH XingLing Sci&Tech.Pharmaceutical Co.,Ltd., Shanghai, 201707 China; 3 State Key Laboratory of Integration and Innovation of Classic Formula and Modern Chinese Medicine, Shanghai University of Traditional Chinese Medicine, Shanghai, 201203 China; 4 National Innovation Platform for Medical Industry-Education Integration, Shanghai University of Traditional Chinese Medicine, Shanghai, 201203 China; 5 Hebei Open University, Shijiazhuang, 050080 Hebei China

**Keywords:** Type 2 diabetes mellitus, Zhimu and Huangbai herb pair decoction, Nanoparticles, Enhancing bioavailability, Comparative pharmacokinetics

## Abstract

Traditional Chinese medicine (TCM) decoctions represent a complex system comprising multiple phases, and their therapeutic effects cannot be fully elucidated solely at the molecular level. The nanophase within TCM decoctions plays a significant role in mediating their pharmacological activities. Zhimu and Huangbai herbal decoction (ZBD), traditionally used to treat type 2 diabetes mellitus (T2DM), is a formulation combining Rhizoma Anemarrhenae and Cortex phellodendri chinensis. Our prior research identified natural nanoparticles (N-ZBD) within ZBD. However, the role of these nanoparticles in ZBD’s anti-T2DM effects and their potential impact on the oral bioavailability of its active components remain unclear. Therefore, this study aimed to examine the anti-T2DM effects of N-ZBD and to assess whether N-ZBD could enhance the bioavailability of its primary active compounds. Initially, we employed a dialysis centrifugation method to isolate the N-ZBD (nanoparticles derived from Zhimu-Baihu Decoction) from ZBD. The separated N-ZBD exhibited an average particle size of 234.4 ± 1.04 nm, a polydispersity index (PDI) of 0.56 ± 0.06, and a zeta potential of − 12 .97 ± 1.46 mV. Then, a type 2 diabetes mellitus (T2DM) rat model was created by administering a low dose of streptozotocin (35 mg/kg) intraperitoneally following 4 weeks on a high-fat diet. After a 7-week treatment period, compared with the model group, the levels of FBG, TC, TG and LDL in MET group, N-ZBD group and ZBD group were significantly reduced, and the morphology of islets was significantly improved. In all phases state of ZBD, N-ZBD had a significant therapeutic effect on T2DM rats, which was comparable to that of ZBD. In order to explore whether N-ZBD has the effect of promoting absorption, we assessed the intestinal absorption properties of N-ZBD, ZBD, and free drugs (mangiferin, timosaponin BII, berberine, phellodendrine, neomangiferin and jatrorrhizine) utilizing a single-pass intestinal perfusion (SPIP) model. Compared to free drugs, N-ZBD enhanced the absorption of active ingredients in the jejunum and ileum. In order to explore whether N-ZBD can improve the bioavailability of active ingredients, a method using ultra-performance liquid chromatography-tandem mass spectrometry (UPLC-MS/MS) was also established to compare the pharmacokinetic profiles of six primary active compounds in plasma after the oral administration of ZBD, N-ZBD and free drugs. The pharmacokinetic profiles of N-ZBD and ZBD in rats were comparable. Notably, N-ZBD exhibited higher *C*_max_, AUC_0-t_, and *T*_1/2_ values for the index compounds compared to free drugs and a reduced plasma clearance rate. In conclusion, N-ZBD is primarily responsible for ZBD’s anti-T2DM effects and significantly enhances the bioavailability of active ingredients, highlighting the essential role of natural nanoparticles in the therapeutic effectiveness of decoctions.

**Supplementary Information:**

The online version contains supplementary material available at 10.1186/s13020-025-01290-z.

## Introduction

Traditional Chinese medicines (TCM) are extensively applied in preventing and treating diverse diseases, attributed to their complex active ingredients and significant therapeutic benefits. Active ingredients have long been isolated from TCM using advanced techniques like preparative liquid chromatography, and these individual compounds are commonly utilized to explore the therapeutic mechanisms of decoctions [21, 31]. However, studies have shown that the bioavailability of purified compounds is often lower than that of decoctions [38]. For instance, the blood concentration of mangiferin was significantly higher in both the *Anemarrhena asphodeloides* Bunge (Zhimu) decoction and the Zhimu-Huangbai decoction (a combination of Zhimu and the cortex of *Phellodendron chinense C.K.*Schneid (Huangbai)) compared to free mangiferin administered at the same dose [13] (All plants name has been checked on 24 February 2025 with http://www.worldfloraonline.org). The AUC of gentiopicroside was significantly reduced after oral administration in its free form compared to that in *Gentiana macrophylla* Pall decoctions [22]. Based on multi-target therapeutic theory [22], the pharmacological effects of natural products are likely due to the synergistic action of multiple constituents rather than a single active compound [36, 40]. Consequently, the interactions between coexisting constituents in decoctions may influence drug absorption upon oral administration.

During decoction, phytochemicals from herbs or plant materials dissolve in boiling water, forming a complex system with multiple phases, such as molecules, nanoaggregates, precipitates, and emulsions [11, 12, 15, 40]. Studies have identified naturally formed colloid-like aggregates in 60 herbal types and 24 traditional Chinese formulations [42]. Investigators have partially investigated these nanoparticles’ formation mechanisms, solubilization, and pharmacological effects. For instance, Yang et al. discovered that nanoparticle formation in the Bai-Hu-Tang decoction was influenced by the prescription’s formulatio [18]. The nano aggregates in the Ge-Gen-Qin-Lian-Tang formulation enhanced baicalin absorption in Caco-2 cells and showed notable antidiabetic effects in diabetic rats induced by STZ [12]. These findings highlight the crucial role that nanoparticles play in decoctions and their substantial Impact on the biological activity of decoctions in clinical treatment.

Herb pairs represent the most basic and straightforward form of multi-herb formulations. The Zhimu-Huangbai pair (ZBD), composed of Zhimu and Huangbai, has long been used in TCM to type 2 diabetes mellitus (T2DM) [35]. Phytochemical studies show that ZBD contains various constituents, including flavonoids [10, 28], alkaloids [3, 8], saponins [14], organic acids [28], and polysaccharide [3]. Active anti-diabetic compounds identified within ZBD include berberine, mangiferin, neo mangiferin, phellodendrine, jatrorrhizine, timosaponin BII, backbone, and polysaccharides [9, 19, 24, 26, 29, 34, 39, 41]. Our preliminary research found that the ZBD forms nanoparticles with a particle size of 225.9 nm. These nanoparticles (N-ZBD) comprise small molecules, polysaccharides, and proteins [17]. They remain stable in the gastrointestinal environment and show some sustained-release effects. In addition, proteins and polysaccharides can influence bioactive compounds’ structural forms and intestinal absorption [16]. Nanoparticles can adsorb bioactive compounds and enhance absorption [6, 32]. These results indicate that nanoparticles in decoction could potentially enhance the therapeutic effects by improving the oral bioavailability of bioactive compounds, although few comprehensive studies have systematically investigated this aspect. Research on naturally self-assembled nanoparticles in decoctions is still relatively limited, with studies only partially addressing nanoparticle isolation [15], characterization [40], solubilization [15], and formation mechanisms [18]. Further investigation is needed to determine whether these self-assembled nanoparticles exhibit pharmacological activity or enhance absorption.

**Fig. 1:**
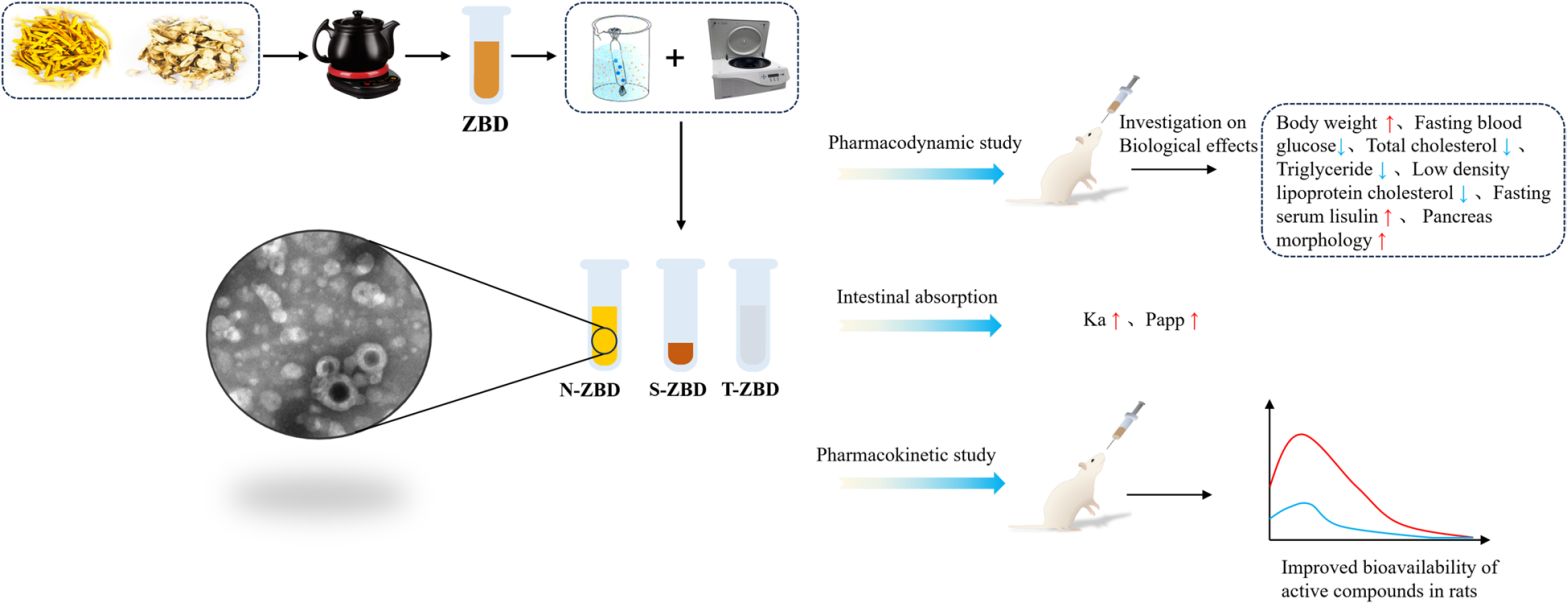
Experimental scheme of the study

This study thus aims to examine the pharmacological differences of ZBD across its various phase states in treating T2DM. By comparing the pharmacokinetics of free compounds and N-ZBD, this research will investigate whether N-ZBD enhances the absorption of active compounds. The experimental scheme is showed in Fig. 1. This research, viewed from a novel supramolecular perspective, proposes that self-assembled nanoparticles may be a fundamental material underlying the combined use of Zhimu and Huangbai. The methods and findings here offer a reference framework for investigating how nanoparticles in other decoctions may affect the efficacy of compound formulations.

## Method

### Chemicals and reagents

**Fig. 2:**
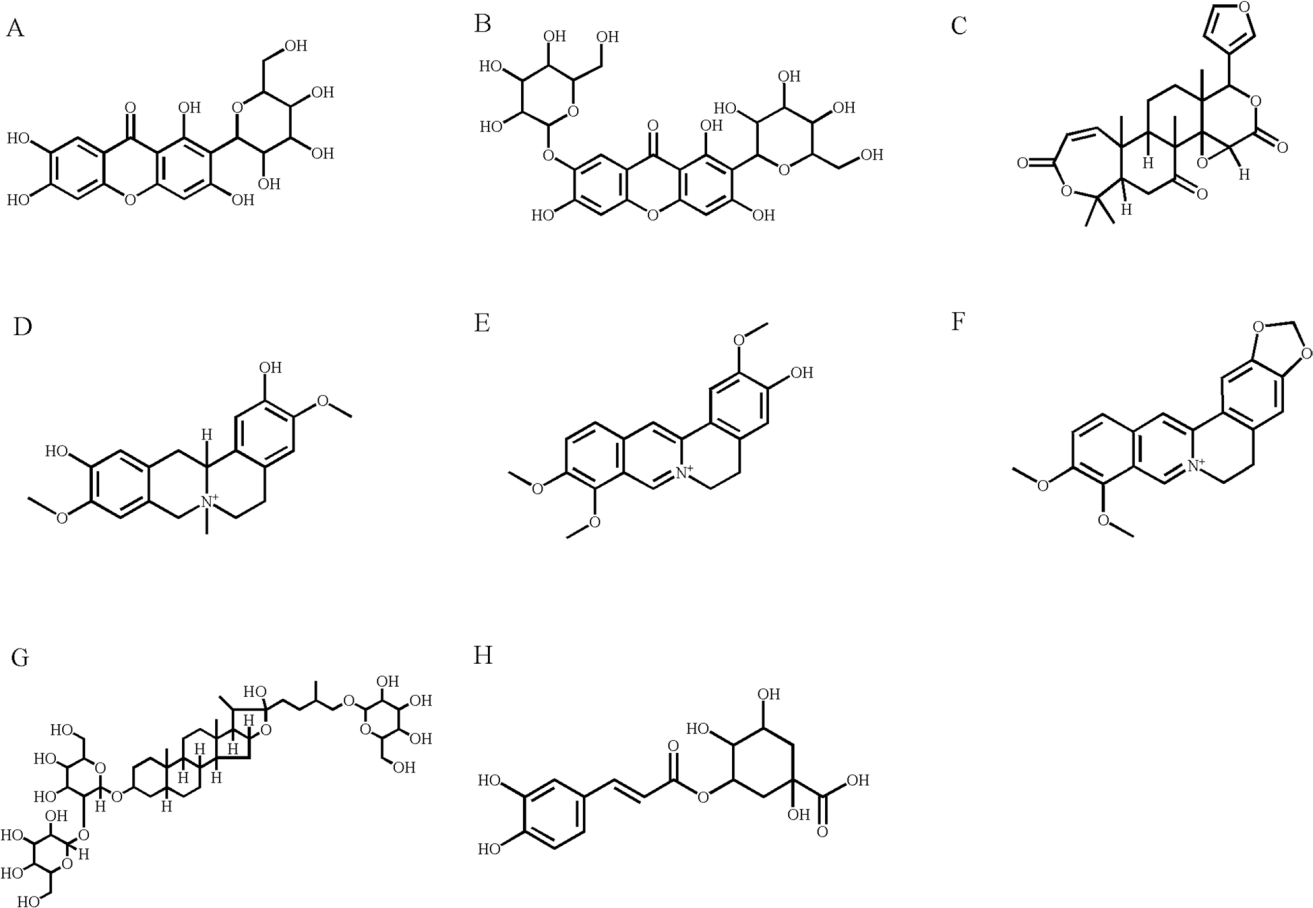
Chemical structures of main pharmacodynamic compounds: mangiferin (**A**), neomangiferin (**B**), obacunone (**C**), phellodendrine (**D**), jatrorrhizine (**E**), berberine (**F**), timosaponin BII (**G**), chlorogenic acid (**H**)

Zhimu (Batch No.: 20210526, Shanghai, China) and Huangbai (Batch No.: 20211007, Shanghai, China) were obtained from Shanghai Kangqiao Traditional Chinese Medicine Co., Ltd. Standards including mangiferin (Fig. 2A), neomangiferin (Fig. 2B), timosaponin BII (Fig. 2G), phellodendrine (Fig. 2D), jatrorrhizine (Fig. 2E), berberine (Fig. 2F), chlorogenic acid (Fig. 2H) and obacunone (Fig. 2C) (≥ 98% purity) were sourced from Shanghai Yuanye Biotechnology Co., Ltd. (Shanghai, China). A 3500 Da dialysis bag was also purchased from Shanghai Yuanye Biotechnology Co., Ltd. (Shanghai, China).

### Preparation of ZBD and separation of the phase states in ZBD

**Fig. 3:**
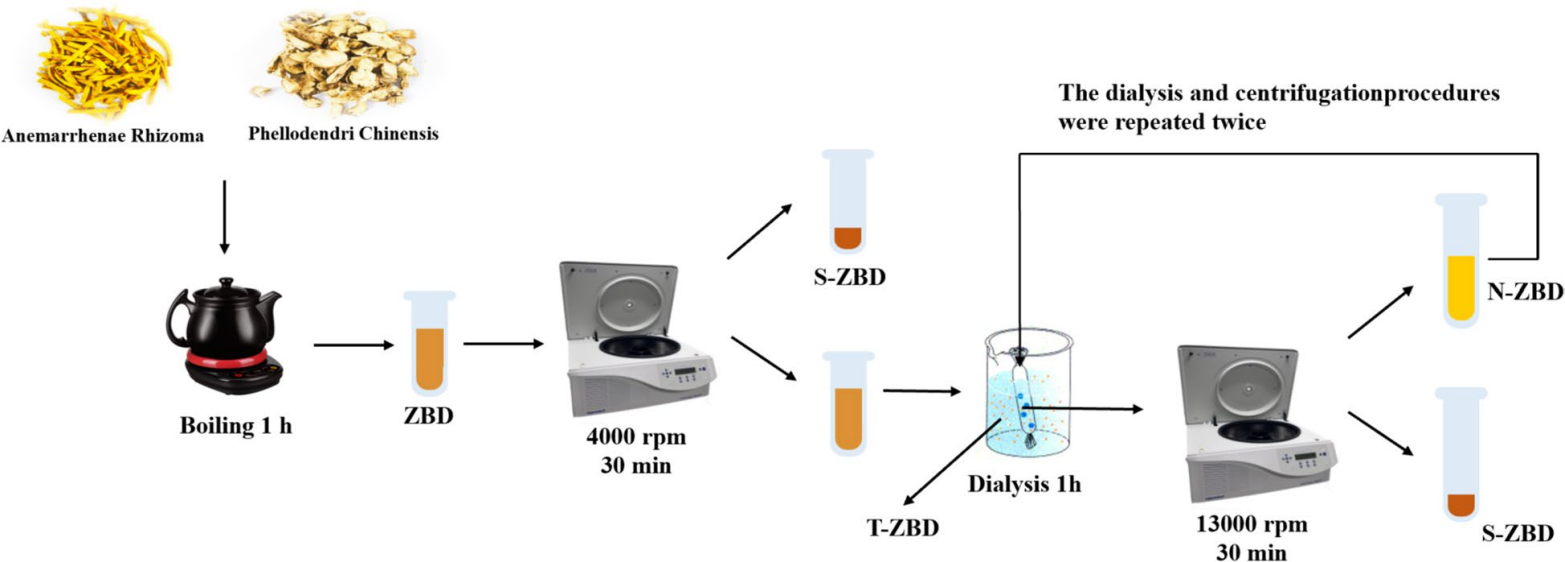
Isolation method for N-ZBD

ZBD was prepared: Zhimu (30 g) and Huangbai (30 g) were immersed in 600 mL of distilled water for 30 min and then boiled for 60 min. Finally, the mixture was filtered through four layers of gauze to obtain ZBD. 50 mL of ZBD was centrifuged at 4000 rpm for 30 min. The supernatant was placed in a tightly sealed dialysis bag, immersed in 500 mL of water, and dialyzed at 37 °C for one hour with gentle agitation (120 rpm). The dialysis bag contents were further centrifuged at 13,000 rpm for 30 min. This dialysis and centrifugation process was repeated twice. As a result of this method, all centrifuged precipitates were collected, yielding the sediment phase (S-ZBD). The outer dialysis liquid represented the true solution phase (T-ZBD), while the liquid inside the dialysis bag was nanoparticles (N-ZBD) [17], as shown in Fig. 3.

### Characterisation of N-ZBD

One drop each of N-ZBD was evenly spread on a copper mesh coated with carbon film. After drying, they were stained with 2.0% phosphotungstic acid for 2 min and dried again. The morphology of the nanoparticles was then observed under transmission electron microscopy (TEM, Tokyo, Japan). The particle size distribution and surface potential of N-ZBD were determined using a Zetasizer Nano ZSE (Malvern Zetasizer Nano-ZS ZEN 3600, Marvin, England) [17].

### UPLC-Q-TOF–MS analysis

An Agilent ZORBAX Eclipse Plus C18 column (2.1 mm × 100 mm, 1.8 μm) was used. Gradient elution was performed with mobile phases consisting of 0.1% formic acid aqueous solution (A) and acetonitrile (B) as follows: 5% B from 0 to 1 min; 5–10% B from 1 to 20 min; 10–21% B from 20 to 35 min; 21–30% B from 35 to 45 min; 30–45% B from 45 to 55 min; and 45–50% B from 55 to 65 min. The column temperature was maintained at 35 °C, the flow rate was 0.3 mL·min⁻^1^, and the injection volume was 2 μL. Both positive and negative ion scan modes were employed. The scan range was set at *m/z* 100–2000. The parameters were as follows: drying gas flow rate, 8 L/min; nebulizer pressure, 35 psi; sheath gas flow rate, 11 L/min; drying gas temperature, 320 °C; sheath gas temperature, 350 °C; ion source voltage, ± 3500 V; declustering potential for primary scanning, 175 V. Mass axis calibration was performed prior to injection to ensure a mass axis error of less than 2 ppm.

The original data were processed by Agilent software Qualitative Analysis 10.0, and both the primary and secondary mass spectrometry data were qualitatively analyzed based on the self-built compound database of the Agilent software Personal Compound Database Library Manager B.08.00. The database was established based on the public database of metabolite information (https://pubchem.ncbi.nlm.nih.gov/; https://old.tcmsp-e.com/tcmsp.php) and literature [23, 25, 30, 33]. Some compounds were also identified with the help of commercial standards analyzed under the same chromatographic conditions.

### Pharmacodynamic evaluation on ZBD, N-ZBD, S-ZBD and T-ZBD

#### Animals and induction of T2DM in rats

Male Sprague–Dawley rats weighing 200–220 g were obtained from Shanghai Sippr-BK Laboratory Animal Co., Ltd. (Shanghai, China). The rats were kept at the Laboratory Animal Center of Shanghai University of Traditional Chinese Medicine under controlled conditions, maintaining an ambient temperature of 22–24 °C and a relative humidity of 60–65%. The rats were allowed access to food and water during the study. All animal experiments received approval from the Animal Ethics Committee of the Experimental Animal Centre at Shanghai University of Traditional Chinese Medicine (Approval No. PZSHUTCM220725022).

T2DM was induced in rats using a combination of a high-glucose, high-fat diet and streptozotocin injection, following previously described method [4]. In brief, rats were given a standard diet for 3 days, followed by a high-glucose, high-fat diet for 4 weeks, and finally an injection of 35 mg∙kg^−1^ streptozotocin [1]. To confirm the successful establishment of the type 2 diabetes model, fasting blood glucose (FBG) levels were assessed 72 h after injection.

#### Animal grouping and administration

Successfully modeled rats were randomly divided into six groups: model group, positive control (metformin) group, ZBD group, N-ZBD group, T-ZBD group, and S-ZBD group, with six rats per group. Rats in the ZBD, N-ZBD, T-ZBD, and S-ZBD groups received an intragastric administration of 6 g crude drug/kg [5]. N-ZBD was administered using a post-concentration strategy to achieve the desired dosage. The particle size and zeta potential of N-ZBD after concentration and subsequent dilution are shown in Figure S5. ZBD, N-ZBD, and T-ZBD were administered intragastrically in liquid form, while S-ZBD was suspended in distilled water before administration. Metformin hydrochloride tablets were crushed, suspended in distilled water, and administered intragastrically at 200 mg/kg. Rats in the model and blank groups received intragastric normal saline at a volume of 10 mL/kg, administered once daily for 35 days. All groups were provided primary feed and unrestricted water access throughout treatment.

The contents of neomangiferin, chlorogenic acid, phellodendrine, mangiferin, jatrorrhizine, berberine, obacunone, and timosaponin BII in ZBD, S-ZBD, T-ZBD, and N-ZBD were determined using the UPLC and HPLC-ELSD methods established in our previous study; specifically, the concentrations of these eight components in ZBD were 443.36, 198.98, 239.52, 339.92, 8.92, 1691.53, 7.88, and 2084.5 μg/mL, respectively, and when compared to the total contents in ZBD, the relative percentages of the above components in each sample were as follows: in N-ZBD, they were 85.67%, 75.32%, 73.46%, 84.49%, 84.59%, 83.70%, 88.63%, and 86.65%, respectively; in S-ZBD, they were 1.42%, 1.49%, 1.31%, 2.11%, 2.54%, 1.89%, 9.69%, and 1.28%, respectively; and in T-ZBD, they were 12.91%, 23.19%, 25.23%, 13.40%, 12.87%, 14.41%, 1.68%, and 12.07%, respectively.

#### Sample collection

Following the 35-day treatment period, all rats were fasted for 12 h with unlimited water access. After anesthesia, blood samples were taken from the abdominal aorta into coagulation tubes and allowed to sit at room temperature for 1 h. The samples were centrifuged at 3500 rpm for 15 min at 4 °C to separate the serum. The serum was aliquoted into centrifuge tubes and stored at -80℃ for future analysis of indicators. Immediately after blood collection, the rats were dissected, and pancreatic tissues were collected. Blood residues on the tissue surfaces were rinsed with saline, and after blotting dry with filter paper, the tissues were fixed in 4% paraformaldehyde for HE staining and pathological examination.

#### Detection of fasting blood glucose

On days 0, 7, 14, 21, 28, and 35 of treatment, animals in each group were fasted for 12 h while having free access to water. A 100 μL blood sample was collected from the tail tip, and fasting blood glucose (FBG) levels were measured using a portable blood glucose meter.

#### Detection of fasting serum insulin content

Serum insulin levels were measured following the kit instructions precisely.

#### Detection of blood lipid and related indicators

Thaw the frozen serum sample from Sect. 2.4.3 to room temperature, then measure the serum levels of TC, TG, and LDL using an automatic biochemical analyzer.

### Rats in situ single-pass intestinal perfusion (SPIP) model studies

Eighteen male Sprague–Dawley rats were randomly divided into three groups: ZBD group, N-ZBD group, and free drug group, with six rats in each group. Before the experiment, the rats were fasted for 24 h with unrestricted water access. Following anesthesia, they were secured on the operating table. A 2–3 cm incision was made along the midline of the rat’s abdomen. Small cuts were then made at the jejunum and ileum ends, where rubber tubes were inserted and secured with surgical sutures. The abdominal opening was covered with saline-moistened gauze, and body temperature was maintained using an infrared lamp to prevent dehydration and heat loss. The ligation sites for the jejunum and ileum were defined as follows: the jejunum was located 15 cm from the pylorus, extending 10 cm downward, while the ileum was 10 cm below a point 20 cm above the cecum. ZBD and N-ZBD were each diluted 1:1 with K-R buffer. The free drug was prepared to match the concentration of the 8 compounds in N-ZBD and then diluted 1:1 with K-R buffer. Before the experiment, flush the intestines with normal saline to clear their contents. Then, perfuse with a drug-containing solution at 37 °C, flowing at 0.4 mL/min for 30 min to equalize drug concentrations at the inlet and outlet. Next, reduce the flow rate to 0.2 mL/min. At the inlet, use a small, pre-weighed bottle containing the perfusion solution, and at the outlet, collect samples in an empty pre-weighed bottle. Samples were collected every 15 min, totaling four times. Following perfusion, the mass differences of the inlet vial (Δ*m*_*in*_) and outlet vial (Δ*m*_*out*_) were accurately measured. The length (L) and inner diameter (R) of the jejunum and ileum segments within the perfused section were also precisely recorded. Collect the supernatant, add methanol in a 1:1 volume ratio, vortex thoroughly, and centrifuge at 13,000 rpm before UPLC analysis. Calculate the absorption rate constant (*K*_*a*_) and the apparent permeability coefficient (*P*_*app*_) using the formulas provided below:

$$K_{a} \, = \,({1} - C_{out} \Delta m_{out} /C_{in} \Delta m_{in} )Q/V$$

$$P_{{app}} \, = \,[ - Q\cdot{\text{ln}}(C_{{out}} \Delta m_{{out}} /C_{{in}} \Delta m_{{in}} )]{\text{ }}/{\text{2}}\pi {\text{R}}L$$

*C*_in_ and *C*_out_: czion solutions; Δ*m*_out_ and Δ*m*_in_: mass differences of inlet and outlet perfusion solution vials; *Q*: perfusion rate; *l*: length of the perfused intestinal segment; R: inner diameter of the perfused intestinal segment; *V*: volume of the perfused intestinal segment.

### Pharmacokinetic study

#### Mass spectrometry system for qualitative assessment

An Agilent 6460 MS/MS system (Agilent Technologies, Santa Clara, CA, USA) was coupled with an Agilent 1290 UPLC system (Agilent Technologies, Santa Clara, CA, USA) for the quantification of analytes using ESI + ionization mode. The capillary voltage was set to 4000 V, with a gas flow rate of 11 L/min. The nebulizer gas pressure was maintained at 15 psi, and the gas temperature was set to 300 °C. Gradient elution was carried out at a flow rate of 0.4 mL/min, using 0.1% (v/v) formic acid as solvent A and acetonitrile as solvent B on an Agilent EC-C18 column (2.1 mm × 100 mm, 1.8 µm) to enhance results. The gradient program was as follows: 90% A from 0 to 1 min, 90–64% A from 1 to 3 min, 64–52% A from 3 to 4 min, 52–10% A from 4 to 5 min, 10% A from 5 to 6 min, 10–90% A from 6 to 6.1 min, and 90% A from 6.1 to 8 min. The injection volume was set at 5 μL. The mass spectrometric parameters for multiple reaction monitoring (MRM) are provided in Table 1.

**Table 1:** Multiple reaction monitoring parameters of mass spectrometer detector

Compounds	RT	MS1	MS2	Dwell	Frag (V)	CE (V)
Mangiferin	2.257	423.0	273.0	100	115	25
Timosaponin BII	3.674	943.5	925.3	100	380	64
Berberine	4.562	335.9	319.9	100	155	32
Phellodendrine	2.502	342.0	192.0	100	115	23
Neomangiferin	1.583	585.2	272.9	100	150	44
Jatrorrhizine	4.593	338.1	322.1	100	140	30
Tetrahydropalmatine (IS)	4.025	356.9	192.0	100	130	28

#### Sample preparation

The internal standard (IS) solution (tetrahydropalmatine, 50 ng/mL) was combined with 50 µL of rat plasma in a 1.5 mL centrifuge tube and mixed for five min. Next, 500 µL of methanol was added, followed by vortexing for 20 min and centrifugation at 4 °C for 10 min at 13,000 rpm. The supernatant was transferred to a new 1.5 mL centrifuge tube and gently evaporated under a nitrogen stream at 37 °C. It was then reconstituted with 50 µL of 10% methanol. After centrifugation at 4 °C for 10 min at 13,000 rpm, 5 µL of the supernatant was collected and injected into the column for analysis.

#### Method validation

Method validation parameters included specificity, Linearity, precision, accuracy, matrix effects, recovery, and stability.

#### Specificity

Specificity was evaluated by comparing the MRM chromatograms of blank rat plasma, blank plasma spiked with the six target compounds and IS, and plasma samples from rats 2 h after oral administration of ZBD.

#### Linearity

Blank plasma samples were examined at eight different concentration levels of the analytes. A standard curve was created through linear regression, plotting the ratio of the analyte peak area to the internal standard (Y) on the vertical axis against the mass concentration of each analyte (X) on the horizontal axis. The limit of quantification (LOQ) was determined based on a signal-to-noise ratio of S/N ≥ 10.

#### Precision and accuracy

Precision and accuracy were evaluated by analyzing six replicates of quality control (QC) samples at low, medium, and high concentrations on the same day and across three consecutive days. Based on theoretical concentrations and observed values, relative error (RE, %) and relative standard deviation (RSD, %) were calculated.

#### Extraction recovery and matrix effect

Extraction recovery was determined by comparing the peak area ratios of the analyte to internal standard in blank plasma samples spiked before and after extraction at low, medium, and high concentrations. The matrix effect was evaluated at three QC levels by contrasting the peak areas of extracted blank plasma samples containing the analyte with those of the pure standard solutions.

#### Stability

The stability of the six analytes in rat plasma was assessed using five sets of QC samples at low, medium, and high concentration levels under various conditions. Short-term stability assessments evaluated plasma samples stored in the injector at 4 °C for 24 h and subjected to three freeze–thaw cycles at − 20 °C. Extended stability was evaluated by storing plasma samples at − 80 °C for 30 days.

#### Application to pharmacokinetic analysis

SD rats were randomly divided into three groups: ZBD, N-ZBD, and free-drug, with six rats in each. The ZBD and N-ZBD groups received 6 g/kg of raw herb oral dose. Neomangiferin, mangiferin, berberine, phellodendrine, and jatrorrhizine levels in ZBD were measured by UPLC, while timosaponin BII content was assessed using HPLC-ELSD. The free-drug group received the same dose as the N-ZBD group. Blood samples (0.2 mL) were obtained from the ophthalmic venous plexus at 0.083, 0.25, 0.5, 1, 2, 4, 6, 8, 12, and 24 h post-administration into heparinized centrifuge tubes. Samples were centrifuged at 4 °C (4000 rpm, 10 min), and the supernatant was stored at − 80 °C in 1.5 mL tubes until analysis.

### Statistical analysis

Data are shown as mean ± SD, with group differences evaluated using one-way ANOVA, setting statistical significance at *p* < 0.05. Key pharmacokinetic parameters, including *C*_max_ (peak plasma concentration), *T*_1/2_ (terminal half-life), *T*_max_ (time to reach peak concentration), AUC_0-t_ (area under the curve from zero to time t), AUC_0-∞_ (area under the curve from zero to infinity), CLz/F (clearance adjusted for bioavailability), and MRT_0-t_ (mean residence time from zero to time t), were determined using DAS 2.0 software. Statistical analyses were performed with SPSS 21.0.

## Result

### Separation of nanoparticles from ZBD

**Fig. 4:**
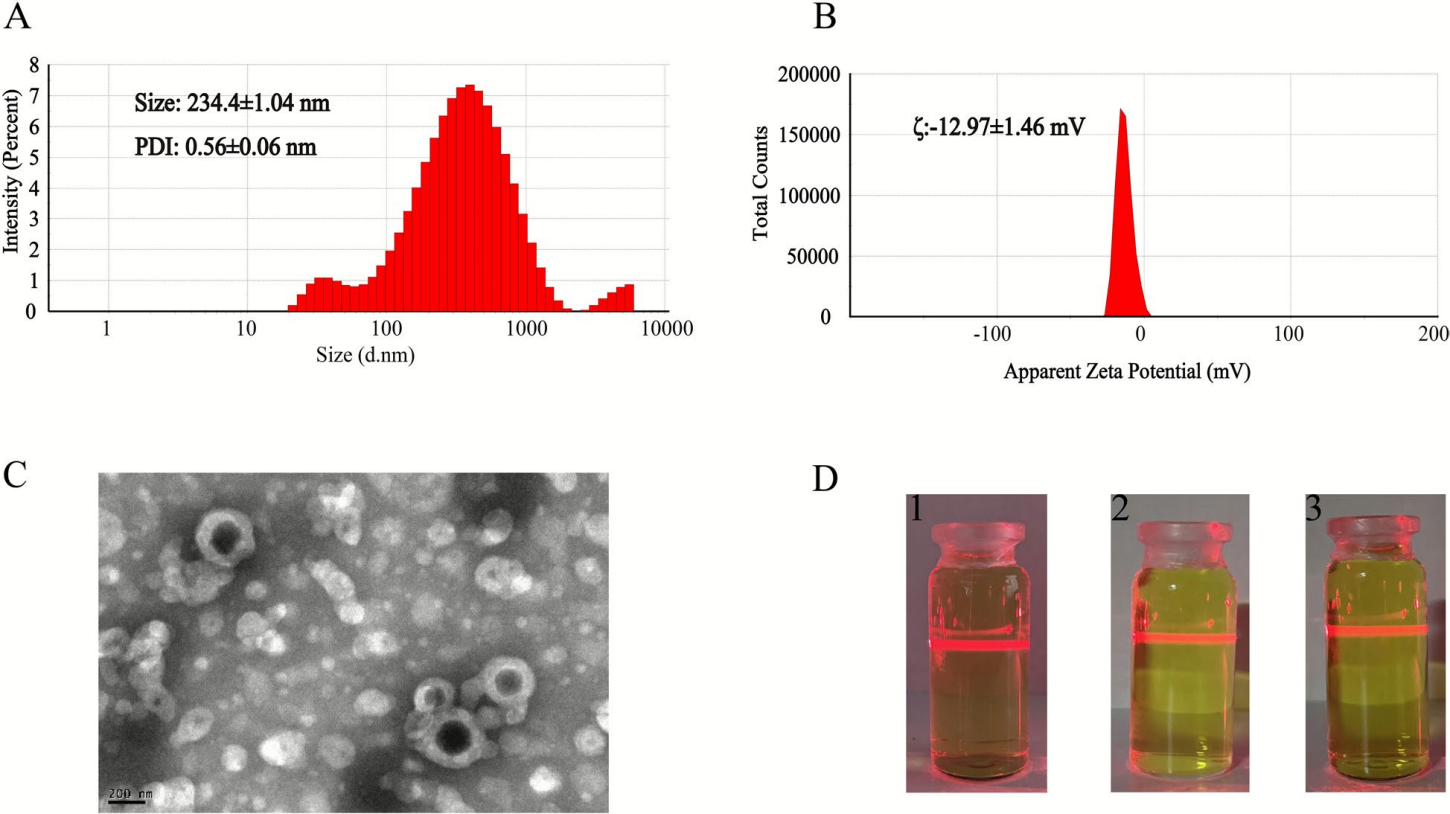
(**A**) Particle size distribution of N-ZBD. (**B**) The zeta potential of N-ZBD. (**C**) TEM images of ZBD. (**D**) Tyndall effects. (1) The tyndall effects of N-ZBD. (2) Tyndall effects in N-ZBD after 12 h incubation in simulated gastric fluid. (3) Tyndall effects in N-ZBD after 12 h incubation in simulated intestinal fluid

N-ZBD was separated by high-speed centrifugation combined with dialysis, a common method for separating nanoparticles from decoctions [15]. Briefly, dialysis removed free drugs from the ZBD, and then high-speed centrifugation removed micron-sized particles to obtain N-ZBD. We mainly investigated the effects of nanoparticle separation methods on the particle size, PDI, zeta potential, and content of each component of N-ZBD, including the molecular weight cut-off of dialysis membranes, centrifugation speed, and dialysis-centrifugation cycles. The results are shown in Tables S1-S3 and Figs. S1-S3. After the final optimized process, the average particle size, PDI, and zeta potential of the obtained N-ZBD were 234.4 ± 1.04 nm, 0.56 ± 0.06, and − 12.97 ± 1.46 mV (Fig. 4A, B), respectively. TEM images also showed the morphology of N-ZBD was composed of black nanoparticles as the inner core and white material as the shell. Our previous studies have demonstrated that the inner core is a self-assembly of small molecular compounds and the outer shell is a polysaccharide [17]. N-ZBD has a significant Tyndall effect (Fig. 4D1), which remains evident after 12 h of incubation in simulated gastric (Fig. 4D2) and simulated intestinal fluids (Fig. 4D3). This showed that N-ZBD could tolerate strong acid and protease decomposition after oral administration.

Our previous study has shown that ZBD is mainly composed of polysaccharides and small molecules, and the main components of ZBD are distributed in N-ZBD [17]. In addition, in order to characterize the chemical constructure of constituents in N-ZBD, UPLC-Q-TOF–MS technique was used, and the representative total ion chromatogram (TIC) of N-ZBD both in negative and positive ion modes were shown in Fig. S4. As a result, A total of 38 components (Table S4) were identified, and 9 of them were verified by chemical standard markers, including chlorogenic acid, neomangiferin, phellodendrine, mangiferin, jatrorrhizine, berberine, timosaponin BII, limonin and obacunone.

### Pharmacodynamic results

#### Impact of various phases on the body weight of T2DM rats

**Fig. 5:**
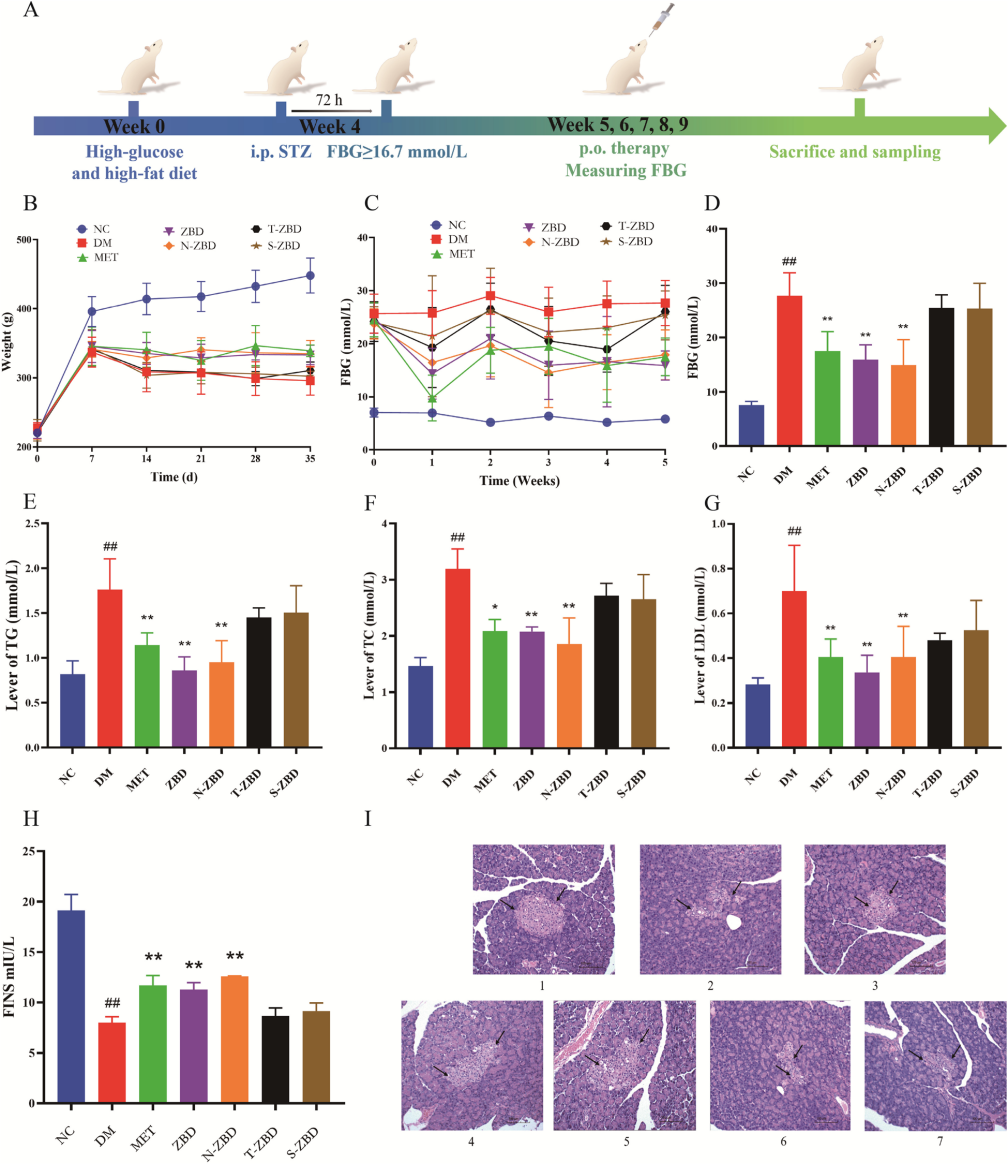
The pharmacodynamic effects of different phases in ZBD on type 2 diabetes in rats. **A** Modeling and administration diagram. **B** Level of body weight within 7 weeks. **C** Level of FBG within 7 weeks. **D** Level of FBG in the 7th week. **E** Level of TG in the 7th week. **F** Level of TC in the 7th week. **G** Level of LDL in the 7th week. **H** Level of FINS. **I** HE staining in pancreas tissue. All data shown were the mean ± standard deviation (SD), with (##) means* p* < 0.01 compared with NC group, with (**) *p* < 0.01 compared with DM group

Weight loss is a common symptom of T2DM. In diabetic patients, lower insulin levels reduce glucose absorption, preventing its entry into cells for energy use. This causes glucose to be excreted in the urine, leading to an energy shortfall. The body metabolizes fats and proteins to offset this deficit, resulting in weight loss. The impacts of the three ZBD phases on body weight in T2DM rats are presented in Fig. 5B. Over the 5-week oral treatment period, the normal group’s body weight consistently rose from 395.67 g to 448.13 g. Conversely, the model group declined, dropping weights from 337.00 g to 295.65 g, substantially lower than the group average (*p* < 0.01). Rats in the MET group showed a marked reduction in weight loss compared to the model group (*p* < 0.01). Likewise, rats in both the ZBD and N-ZBD groups experienced significant weight gain after 5 weeks of treatment compared to the model group (*p* < 0.01). No significant difference in body weight was found between the ZBD and N-ZBD groups (*p* > 0.05). In contrast, after 5 weeks of treatment, the S-ZBD and T-ZBD groups showed no significant change in body weight compared to the model group (*p* > 0.05). These findings suggest that both ZBD and N-ZBD help counteract weight loss, highlighting the critical role of N-ZBD in ZBD’s therapeutic effectiveness.

#### Hypoglycemic effects of different phases in T2DM rats

The schematic diagram of molding and administration is shown in Fig. 5A. Figure 5C, D display the effects of various ZBD phases on blood glucose levels in type 2 diabetic rats. Following successful modeling, fasting blood glucose (FBG) levels in the model group rose significantly (≥ 16.7 mmol/L), while MET notably lowered FBG levels in rats (*p* < 0.01), confirming the successful establishment of the T2DM model. After 35 days of treatment, the ZBD group exhibited significantly lower FBG levels than the model group (*p* < 0.01). Within the ZBD phases, the N-ZBD group demonstrated a notable reduction in FBG levels compared to the model group (*p* < 0.01). Nonetheless, no significant difference in FBG levels was observed between the ZBD and N-ZBD groups, and neither the S-ZBD nor T-ZBD groups showed a significant reduction in FBG levels compared to the model group (*p* > 0.05). These results suggest that N-ZBD exerts a potent hypoglycemic effect comparable to ZBD, while S-ZBD and T-ZBD show no significant hypoglycemic activity. This suggests that N-ZBD is primarily responsible for the hypoglycemic action of ZBD.

#### Hypolipidemic effects of different phases in T2DM rats

Most patients with T2DM show disrupted glucose and lipid metabolism, typically marked by notably higher levels of triglycerides (TG) (Fig. 5E), total cholesterol (TC) (Fig. 5F), and low-density lipoprotein (LDL) (Fig. 5G). Experimental results indicated that the model group displayed significantly higher TG, TC, and LDL levels than the control group (*p* < 0.01), indicating lipid metabolic disorder and successful diabetes model establishment. The positive control, metformin, and ZBD significantly lowered TC, TG, and LDL levels compared to the model group (*p* < 0.01). Of the three ZBD phases, N-ZBD demonstrated the greatest efficacy, similar to that of ZBD, while T-ZBD and S-ZBD showed no significant differences from the model group within the indicators assessed (*p* > 0.05). These findings identify N-ZBD as the active phase of ZBD for addressing lipid metabolism disorders in T2DM rats.

#### HE staining in pancreas tissue

Figure 5I display the histological analysis of HE-stained pancreatic tissues in each rat group. In the NC group, pancreatic acini displayed normal morphology, with well-defined, moderately sized islets, showing no necrosis or inflammatory cell infiltration. In contrast, the DM group exhibited atrophied islets with fewer and smaller islet cells and densely packed nuclei. Compared to the DM group, the MET, ZBD, and N-ZBD groups showed notably reduced islet atrophy, with a greater number of cells and improved cell morphology. However, the S-ZBD and T-ZBD groups did not show significant morphological improvement in islet cells relative to the model group. The Impact of different phases on serum FINS levels in T2DM rats is shown in Fig. 5H. The MET group demonstrated a significant rise in FINS levels compared to the NC group (*p* < 0.01). The MET and ZBD groups also demonstrated significantly higher FINS levels than the DM group (*p* < 0.01). Of the three ZBD phases, N-ZBD demonstrated the highest efficacy, comparable to that of ZBD, whereas T-ZBD and S-ZBD groups exhibited no notable difference from the model group (*p* > 0.05). These findings suggest that ZBD may improve islet tissue morphology and boost insulin secretion in T2DM rats, with N-ZBD exhibiting effects similar to those of ZBD.

### The result of situ single-pass intestinal perfusion (SPIP) studies

**Fig. 6:**
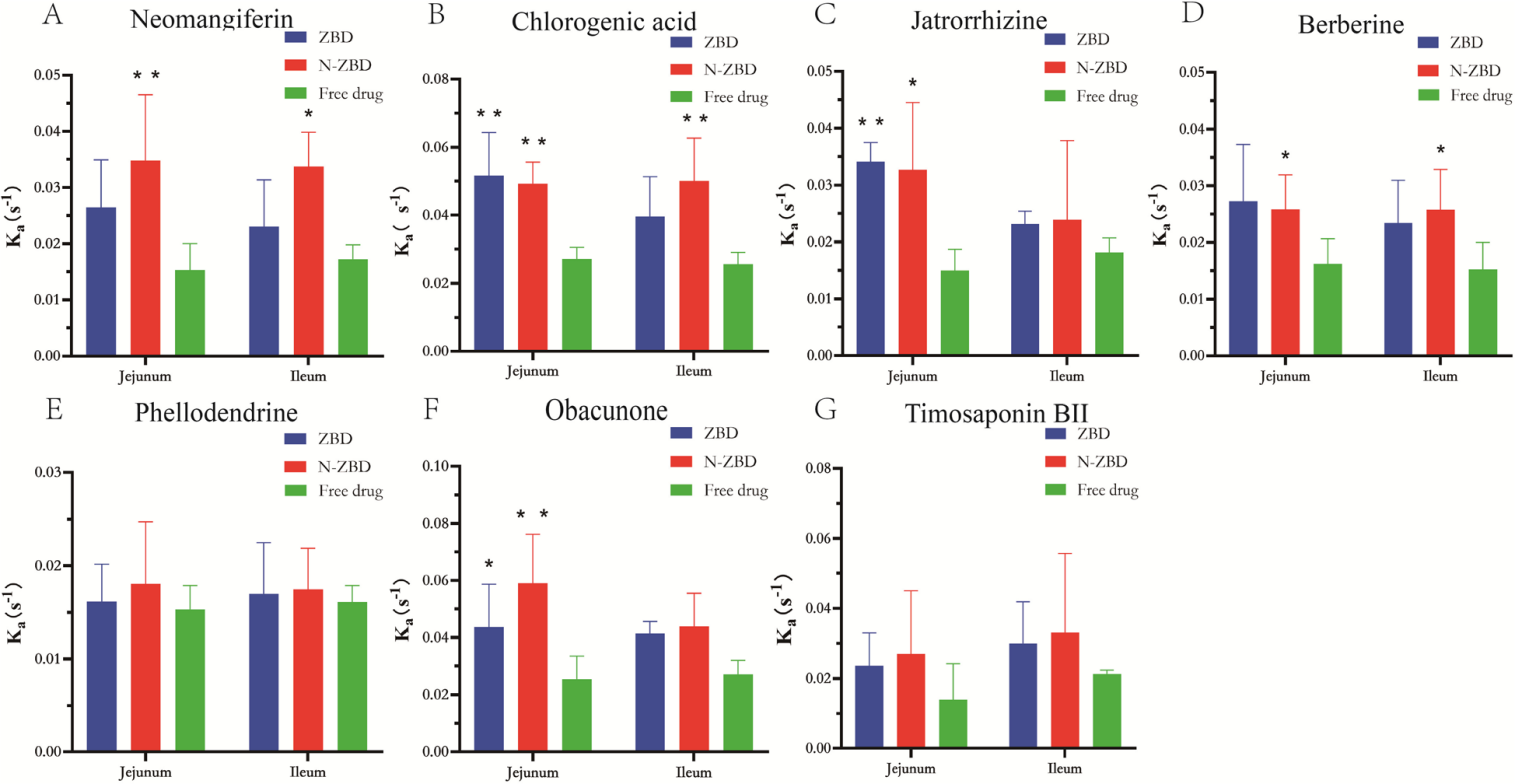
The *K*_*a*_ of ZBD, N-ZBD and free drugs measured by SPIP. (**A**) Neomangiferin; (**B**) Chlorogenic acid; (**C**) Jatrorrhizine; (**D**) Berberine; (**E**) Phellodendrine; (**F**) Obacunone; (**G**) Timosaponin BII

**Fig. 7:**
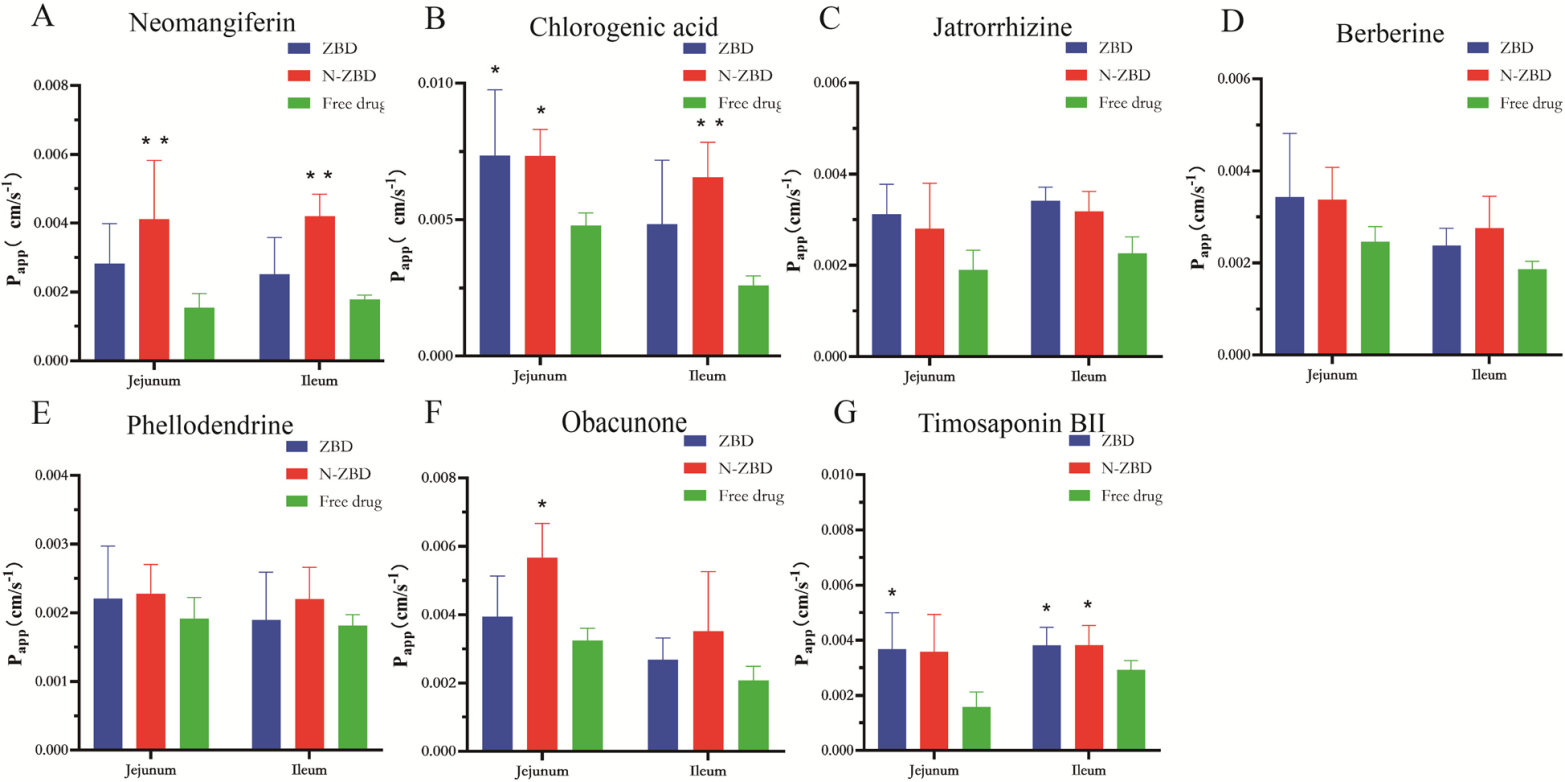
The *P*_*app*_ of ZBD, N-ZBD and free drugs measured by SPIP. (**A**) Neomangiferin; (**B**) Chlorogenic acid; (**C**) Jatrorrhizine; (**D**) Berberine; (**E**) Phellodendrine; (**F**) Obacunone; (**G**) Timosaponin BII

Following oral administration of the decoction, primary absorption occurs in the small intestine. Drug uptake in this region reflects overall post-oral absorption and is key to bioavailability. Thus, examining intestinal absorption of ZBD, N-ZBD, and free drugs can clarify whether N-ZBD improves active compound absorption. Figure 6 and 7 present the experimental outcomes. The *Ka* and *Papp* values for jatrorrhizine, berberine, phellodendrine, and timosaponin BII in the N-ZBD group closely matched those in the ZBD group, likely due to the similar composition of compounds in N-ZBD. In both the jejunum and ileum, *K*_*a*_ and *P*_*app*_ values for neomangiferin, chlorogenic acid, jatrorrhizine, berberine, phellodendrine, and timosaponin BII were higher in the N-ZBD and ZBD groups compared to those in the free drug group. In the jejunum segment, the *Ka* values for neomangiferin, chlorogenic acid, jatrorrhizine, and berberine were markedly higher in the N-ZBD group than in the free drug group (*p* < 0.05).

Similarly, the *P*_*app*_ values for neomangiferin, chlorogenic acid, and berberine levels were also significantly greater than those in the free drug group (*p* < 0.05). In the ileum segment, the Ka values for neomangiferin, chlorogenic acid, and berberine in the N-ZBD group levels were significantly greater than those in the free drug group (*p* < 0.05). Additionally, the *P*_*app*_ values for neomangiferin, chlorogenic acid, and timosaponin BII were significantly elevated compared to the free drug group (*p* < 0.05). These results imply that N-ZBD may enhance the absorption of active compounds in both the jejunum and ileum to varying extents.

### Pharmacokinetic study

In order to compare the pharmacokinetic differences between ZBD, N-ZBD and free drugs, a UPLC-MS/MS method was established for determining six representative drugs in rat plasma, namely mangiferin, timosaponin BII, berberine, phellodendrine, neomangiferin and jatrorrhizine.

#### Method validation

##### Specificity

**Fig. 8:**
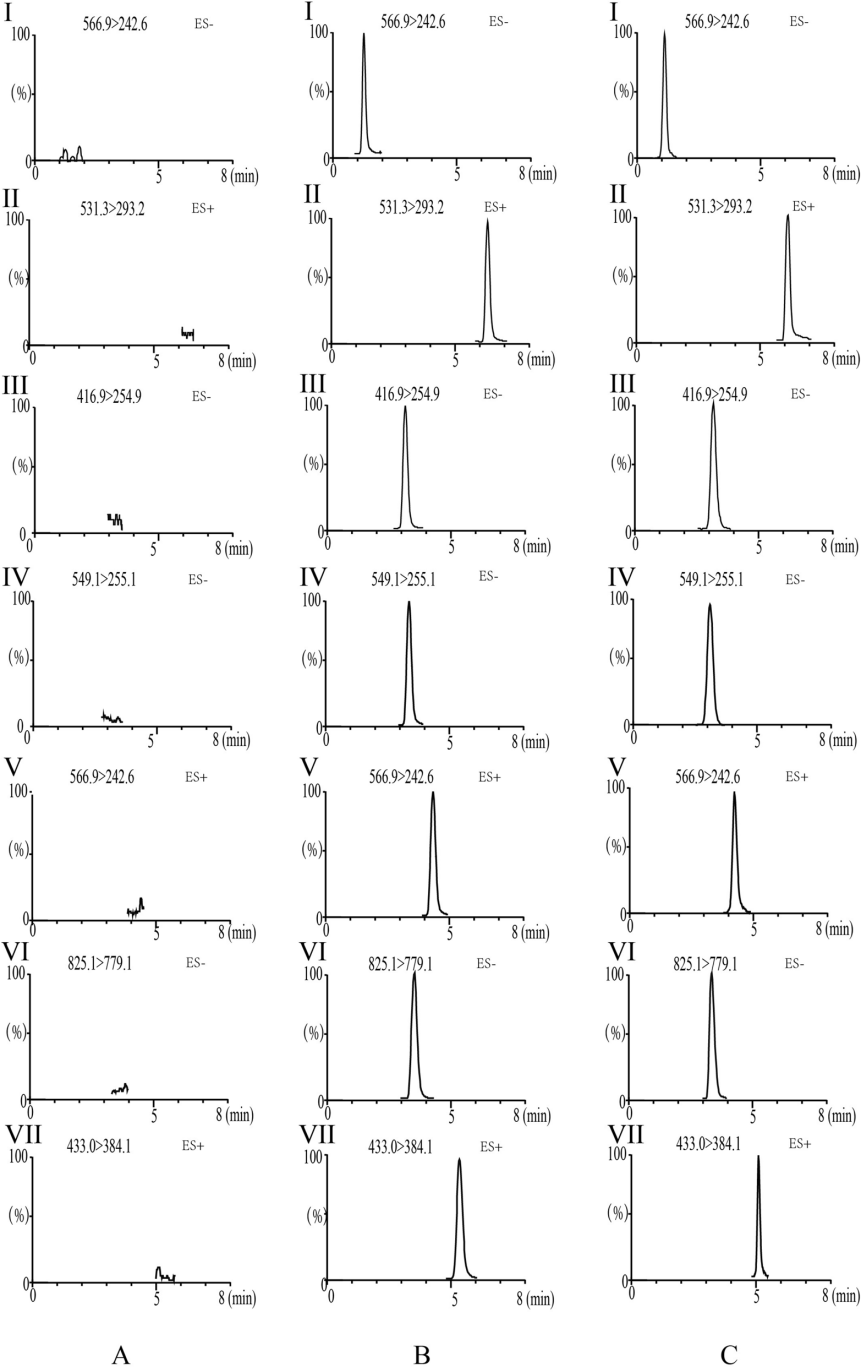
**A** MRM chromatograms of blank plasma samples, **B** blank spiked with combined standard solutions of the six analytes and IS, **C** rat plasma samples at 2 h after oral administration of ZBD. 1, Mangiferin; 2, Timosaponin BII; 3, Berberine; 4, Phellodendrine; 5, Neomangiferin; 6, Jatrorrhizine and 7, Tetrahydropalmatine (IS)

Fig. 8 presents representative MRM chromatograms for blank plasma spiked with analytes and IS the internal standard, and plasma samples collected from rats two h after oral drug administration. No interference was detected between the analytes and the internal standard during the retention time, and peak shapes were well-defined, confirming the method’s strong specificity.

##### Linearity and LLOQ

Table 2 provides the regression equations, linear ranges, and LLOQ values for the standard curves of the six compounds analyzed in rat plasma. The linear ranges for neo mangiferin, phellodendrine, mangiferin, jatrorrhizine, timosaponin BII, and berberine were 0.68–434.14, 0.50–321.00, 0.93–595.00, 0.18–115.64, 1.38–884.70, and 1.43–914.00 ng/mL, respectively, with correlation coefficients (r) exceeding 0.995. This high Linearity supports the method’s suitability for plasma content analysis.

**Table 2:** Calibration curves, LODs and LOQs of 8 analytes

Compound	Regression equation	Correlation coefficient (r)	Linear range (ng/mL)	LLOQ (ng/mL)
Neomangiferin	Y = 5.6598*10-5X + 3.4512*10^–5^	0.9985	0.68–434.14	0.13
Phellodendrine	Y = 2.7257*10-2X + 4.6944*10^–2^	0.9999	0.50–321.00	0.05
Mangiferin	Y = 6.5549*10-5X + 3.3300*10^–4^	0.9942	0.93–595.00	0.31
Jatrorrhizine	Y = 6.2460*10-2X + 1.1380*10^–4^	0.9983	0.18–115.64	0.14
Timosaponin BII	Y = 5.1830*10-5X + 1.4249*10^–4^	0.9949	1.38–884.70	0.01
Berberine	Y = 6.2550*10-3X + 2.7282*10^–1^	0.9985	1.43–914.00	0.01

##### Precision and reliability

Table 3 provides the precision data for the six analytes, covering both intra-day and inter-day measurements. The intra-day precision (RSD) ranged from 0.44% to 13.25%, with accuracy (RE) between − 6.41% and 8.75%. For inter-day measurements, RSD ranged from 0.73% to 14.70%, and RE was between -12.91% and 11.90%. All values satisfied the acceptance criteria, demonstrating that the developed method is reliable, precise, and appropriate for the quantitative analysis of the six analytes.

**Table 3:** Precision and accuracy of 6 compounds in rat plasma (n = 6)

Compound	Concentration (ng/mL)		Intra-day			Inter-day		
			Mean	RSD (%)	RE (%)	Mean	RSD (%)	RE (%)
Neomangiferin		13.57	13.71	13.25	1.02	15.72	14.70	0.05
		54.27	55.05	7.41	1.44	50.27	8.26	− 7.37
		217.07	224.17	6.65	3.27	212.46	8.38	− 2.13
Phellodendrine		10.03	10.71	1.79	6.81	8.74	2.46	− 12.91
		40.13	42.52	5.58	5.96	37.280	6.89	− 7.09
		160.50	150.21	4.71	− 6.41	152.33	4.93	− 5.09
Mangiferin		14.04	13.71	13.25	− 2.41	15.68	12.16	11.66
		56.18	55.05	2.80	− 2	56.19	3.70	0.03
		224.70	224.17	6.65	− 0.24	231.26	8.25	2.92
Jatrorrhizine		8.04	3.45	0.44	− 4.57	3.72	3.65	2.82
		168.08	14.32	1.75	− 0.91	15.68	4.15	8.47
		336.15	54.99	0.74	− 4.9	54.30	0.73	− 6.09
Timosaponin BII		6.31	24.55	6.14	8.75	25.26	6.14	11.90
		126.16	92.34	0.50	2.25	96.98	7.15	7.39
		252.33	346.75	3.09	− 4.01	351.26	5.23	− 2.76
Berberine		28.56	27.45	4.26	− 3.89	26.15	6.28	− 8.45
		114.25	116.24	2.35	1.74	118.15	2.12	3.41
		457.00	462.32	6.26	1.16	463.26	2.69	1.37

##### Extraction recovery and matrix effect

Table 4 presents the extraction recoveries and matrix effects for the six compounds at three different QC levels. Recovery rates ranged from 47.34% to 107.25%, while matrix effects varied between 89.26% and 108.82%. These results confirm that the method’s analytical recovery and matrix effects are within acceptable limits.

**Table 4:** Matrix effect and recovery of 6 compounds from rat plasma (n = 6)

Compound	Recovery (%)			Matrix effect (%)		
	Low	Medium	High	Low	Medium	High
Neomangiferin	100.32 ± 13.34	85.50 ± 1.94	75.98 ± 2.77	104.32 ± 2.59	108.07 ± 5.61	106.85 ± 9.04
Phellodendrine	107.25 ± 9.40	103.72 ± 1.98	96.01 ± 9.44	107.99 ± 0.82	100.21 ± 0.70	98.40 ± 10.45
Mangiferin	59.26 ± 4.10	46.95 ± 2.13	47.34 ± 5.89	102.78 ± 6.93	90.58 ± 2.56	101.46 ± 9.56
Jatrorrhizine	96.58 ± 3.07	88.23 ± 3.44	95.65 ± 6.09	107.82 ± 2.09	99.55 ± 0.84	89.26 ± 2.77
Timosaponin BII	104.54 ± 3.19	105.50 ± 4.67	95.04 ± 10.34	101.86 ± 6.10	108.82 ± 4.65	101.64 ± 8.61
Berberine	100.62 ± 1.58	100.22 ± 2.57	95.15 ± 9.76	105.27 ± 6.24	105.08 ± 4.92	102.20 ± 6.93

##### Stability

Table 5 results indicate that the six compounds in rat plasma maintained stability and met analytical standards after 24 h at 4 °C and three freeze–thaw cycles.

**Table 5:** Stability of six compounds in rat plasma at three QC levels (n = 6)

Compound	Concentration (ng/mL)	Stability during 24 h at 4 ℃		Three freeze–thaw cycles	
		RSD (%)	RE (%)	RSD (%)	RE (%)
Neomangiferin	13.567	14.09	97.54	14.30	92.50
	54.268	10.44	100.64	10.29	100.15
	217.070	8.96	94.89	3.18	100.31
Phellodendrine	10.031	8.17	101.89	10.22	110.37
	40.125	4.80	99.92	6.49	111.64
	160.500	4.61	110.07	8.57	112.59
Mangiferin	14.044	8.07	87.04	11.32	94.85
	56.175	6.22	92.70	6.19	89.74
	224.700	6.91	96.06	6.40	92.97
Jatrorrhizine	3.613	3.76	86.34	8.57	85.01
	14.455	6.56	96.16	6.15	92.38
	57.820	4.40	94.54	6.32	94.11
Timosaponin BII	22.577	8.33	89.47	9.67	87.30
	90.306	9.47	91.42	8.36	87.38
	361.224	10.17	100.28	12.93	99.86
Berberine	28.563	5.83	98.56	8.27	98.17
	114.250	10.98	99.25	9.16	93.11
	457.000	10.19	103.64	10.12	92.07

#### Comparative analysis of pharmacokinetic results in rats

**Fig. 9:**
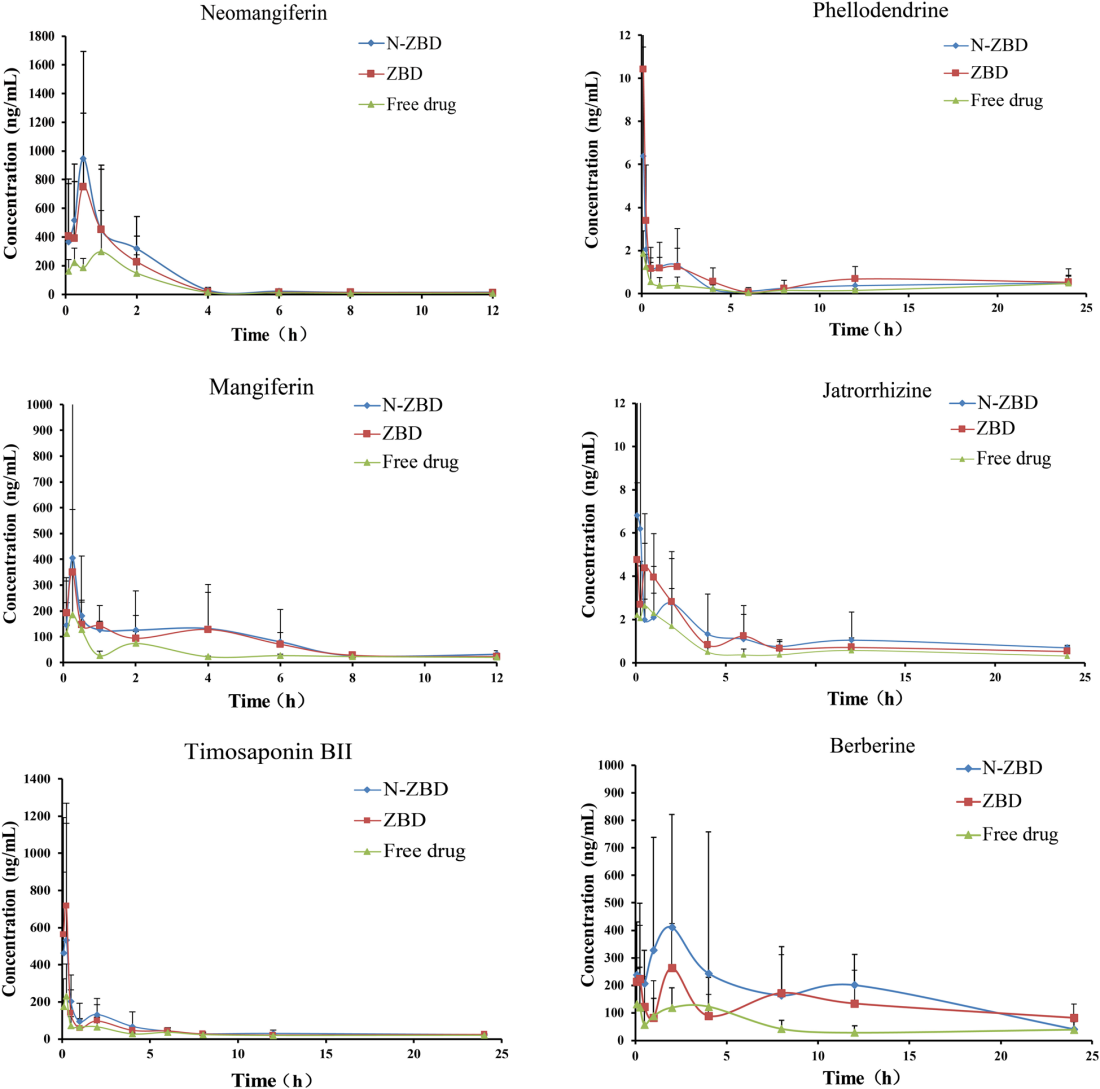
Mean plasma concentration–time curves of six major bioactive compounds after the oral administration of N-ZBD, ZBD and free drugs. The inserted figures show the initial 4 h profiles for the analytes

Pharmacokinetics were evaluated following administration via the oral route of ZBD, N-ZBD, and unbound drugs analyzed by UPLC-MS/MS. Figure 9 displays the average concentration–time profiles for the substances in the plasma of rats from the ZBD, N-ZBD, and free drug groups. Pharmacokinetic parameters, such as *T*_1/2_, *T*_max_, *C*_max_, AUC_0-t_, AUC_0-∞_, CLz/F and MRT_0-t_, were determined with DAS 2.0 software, as presented in Table 6. The plasma concentrations of mangiferin and neo mangiferin at 24 h fell below the limit of quantification, making accurate detection challenging. Consequently, only the drug-time curve for the first 12 h was plotted.

**Table 6:** Pharmacokinetic parameters of six compounds in male SD rats after oral administration of N-ZBD, ZBD and free drugs (mean ± SD, n = 6)

Compound	Groups	*T*_1/2_ (h)	*T*_max_ (h)	*C*_max_ (ng/mL)	AUC_0-t_ (ng/mL*h)	AUC_0-∞_(ng/mL*h)	CLz/F (mL/h/kg)	MRT_0-t_ (h)
Berberine	N-ZBD	6.44 ± 4.55	2.48 ± 2.77	770.29 ± 408.37*	3643.12 ± 1241.03**	6267.20 ± 2866.73**	5.14 ± 1.81**	8.33 ± 2.65
	ZBD	8.79 ± 5.30	4.35 ± 4.90	376.64 ± 158.58	2940.00 ± 970.77**	6145.90 ± 4877.55**	7.45 ± 3.49**	10.54 ± 2.53**
	Free drugs	4.50 ± 2.65	2.33 ± 1.73	236.34 ± 96.63	1054.50 ± 339.78	1439.89 ± 888.15	23.97 ± 9.65	6.10 ± 1.15
Mangiferin	N-ZBD	14.55 ± 4.15	1.42 ± 1.62	439.66 ± 201.80	1480.21 ± 652.17**	2237.23 ± 643.90**	2.57 ± 0.96**	7.71 ± 1.86
	ZBD	15.25 ± 12.66	0.99 ± 1.49	415.61 ± 178.25	1247.54 ± 344.93**	1859.15 ± 655.63**	3.75 ± 1.43**	7.66 ± 1.03
	Free drugs	7.74 ± 3.91	0.36 ± 0.13	241.02 ± 161.47	586.84 ± 89.50	806.31 ± 488.54	7.82 ± 2.73	6.52 ± 0.98
Neomangiferin	N-ZBD	8.43 ± 6.21	0.71 ± 0.27	981.75 ± 711.30*	1607.29 ± 1030.99*	2296.34 ± 1652.12*	4.55 ± 2.77*	3.41 ± 1.56
	ZBD	8.01 ± 4.86	0.27 ± 0.17	996.07 ± 373.48	1300.20 ± 697.45	1372.74 ± 709.56	7.77 ± 4.19	2.89 ± 0.83
	Free drugs	6.78 ± 1.13	0.54 ± 0.34	365.77 ± 259.95	715.25 ± 349.16	731.57 ± 347.24	11.55 ± 4.91	3.13 ± 1.03
Timosaponin BII	N-ZBD	9.69 ± 5.47	0.66 ± 0.66	645.98 ± 584.69*	1151.07 ± 295.78**	1718.03 ± 1166.15**	28.09 ± 11.24**	6.95 ± 2.56
	ZBD	8.30 ± 3.38	0.18 ± 0.09	824.35 ± 605.28	1032.60 ± 301.36**	1133.49 ± 292.42	45.21 ± 13.19**	7.31 ± 2.12
	Free drugs	7.00 ± 1.27	0.61 ± 0.68	240.23 ± 165.78	567.81 ± 131.62	610.34 ± 154.66	66.12 ± 14.92	5.93 ± 0.68
Phellodendrine	N-ZBD	4.11 ± 1.49	0.36 ± 0.72	6.49 ± 4.96	11.50 ± 2.32**	19.05 ± 13.99**	2366.55 ± 829.33**	10.20 ± 4.35
	ZBD	4.29 ± 2.77	4.29 ± 2.77	10.78 ± 5.72**	15.57 ± 5.62**	26.14 ± 8.73**	1402.56 ± 963.37**	9.65 ± 2.23
	Free drugs	3.64 ± 1.87	3.64 ± 1.87	2.03 ± 1.05	2.19 ± 1.96	2.25 ± 2.00	8420.25 ± 1225.92	4.38 ± 8.21
Jatrorrhizine	N-ZBD	5.23 ± 1.97	0.54 ± 0.72	8.85 ± 11.97	27.73 ± 23.01	42.62 ± 29.79	4.19 ± 3.46	9.39 ± 2.26
	ZBD	6.27 ± 2.62*	0.67 ± 0.67	7.31 ± 1.61*	23.89 ± 5.54	34.35 ± 19.43	5.17 ± 2.40	7.30 ± 1.43
	Free drugs	3.73 ± 1.37	2.33 ± 4.31	4.32 ± 2.88	9.69 ± 13.85	21.31 ± 27.19	7.33 ± 9.94	4.67 ± 4.53

*ANOVA* test was used to calculate the significance of the differences, *p < 0.05 which compared with the free drugs; **p < 0.01 which compared with the free drugs.

The results indicated that after the oral administration of N-ZBD and ZBD to rats, all six compounds displayed comparable pharmacokinetic curve characteristics, including similar peak times and peak concentrations. Notably, mangiferin in N-ZBD comprised 84.49% of that in ZBD, yet both formulations showed similar blood concentrations post-administration. Similarly, neomangiferin and berberine in N-ZBD comprised 85.67% and 83.70% of the levels found in ZBD, respectively; however, the peak plasma concentration in rats after oral administration of N-ZBD was higher than that of ZBD. At all time points, the plasma concentrations of the six compounds in the free drug group were lower than those in both N-ZBD and ZBD.

Pharmacokinetic parameters, including *T*_1/2_, *T*_max_, *C*_max_, AUC_0-t_, AUC_0-∞_, CLz/F, and MRT_0-∞_, were determined using DAS2.0 software. The results indicated that the six compounds showed comparable pharmacokinetic parameter values in the N-ZBD and ZBD groups. This similarity may arise from most of the six compounds in ZBD being present in N-ZBD, leading to analogous pharmacokinetic behaviors. In contrast, the pharmacokinetic profiles of N-ZBD and free drugs were markedly different. In the N-ZBD group, the *T*_1/2_ values for berberine, mangiferin, neomangiferin, timosaponin BII, phellodendrine, and jatrorrhizine were extended by 43.10%, 87.98%, 24.34%, 12.90%, and 40.21%, respectively, compared to free drugs. Additionally, the *C*_max_ of neomangiferin and timosaponin BII in the N-ZBD group significantly increased (*p* < 0.05). The AUC_0-t_ values for berberine, mangiferin, neo mangiferin, timosaponin BII, phellodendrine, and jatrorrhizine in the N-ZBD group rose by 245.48%, 152.23%, 124.72%, 102.72%, 425.11%, and 186.17%, respectively. Furthermore, the AUC_0-t_ for berberine, mangiferin, neomangiferin, and timosaponin BII in the N-ZBD group was significantly higher than in the free drug group (*p* < 0.05). The CLz/F values for berberine, mangiferin, neomangiferin, timosaponin BII, and phellodendrine in the N-ZBD group were significantly lower than those in the free drug group (*p* < 0.05). Compared to the free drug group, all six compounds in the N-ZBD group displayed pharmacokinetic characteristics akin to sustained-release formulations following oral administration in rats. Overall, N-ZBD markedly improved the oral bioavailability of the active compounds.

## Discussion

ZBD has traditionally been used in Chinese medicine to treat T2DM [20, 35]. Its application in the comprehensive management of T2DM enhances patients’ quality of life and minimizes the side effects associated with chemical drugs. By means of phase separation of representative traditional Chinese medicine decoctions, it is beneficial to search for the “effective phase” of decoctions and reveal the pharmacological principles underlying the effective effects of traditional Chinese medicine decoctions through studying the contents of active compounds, the physical structures of compound particles and their pharmacological effects under different phase states. As in our previous article [17], we successfully transitioned ZBD into a true solution phase, nanophase, and sediment phase within the solution dispersion system and investigated the gastrointestinal stability, drug release, and formation mechanisms of N-ZBD. The results indicate that N-ZBD remains stable in the gastrointestinal environment, and its formation mechanism involves the self-assembly of small molecules and interactions between small molecules and polysaccharides. However, the Impact of nanoparticles in ZBD on its anti-T2DM effect and their potential influence on the oral bioavailability of active compounds still need to be clarified, and we need comprehensive descriptions of these aspects. Therefore, this study aimed to investigate the anti-type 2 diabetes mellitus effects of N-ZBD and to determine whether N-ZBD enhances the bioavailability of the primary active compounds.

In the animal experiments of this study, the corresponding decoctions were prepared using traditional Chinese medicine decoction pieces of the same batch. Under the condition of 25 °C, the same operator separated each phase state in sequence according to the same steps within 8 h. Although it took us a lot of time and energy every day, this procedure effectively reduced the interferences related to the stability of samples. By comparing the differences in the pharmacodynamic effects of different phase states, the aim is to identify the “effective phase state” of ZBD in the treatment of type 2 diabetes mellitus (T2DM). Pharmacodynamic results indicated that FBG, TC, TG, and LDL levels were significantly reduced in the metformin, N-ZBD, and ZBD groups compared to the model group. Additionally, the indicators in the N-ZBD group were comparable to those in the ZBD group. Nonetheless, no significant differences were observed in the indicators for the S-ZBD and T-ZBD groups compared to the model group. Compared to the DM group, the MET, ZBD, and N-ZBD groups have exhibited significantly reduced islet atrophy, increased islet cells, and improved islet cell morphology. In contrast, the S-ZBD and T-ZBD groups showed no significant improvement in islet cell morphology relative to the model group. These results indicate that N-ZBD has a notable therapeutic effect on T2DM rats, comparable to ZBD.

Generally, nanoparticles smaller than 500 nm with a neutral or negative surface charge enhance mucus penetration, closely linked to intestinal absorption [37]. Consequently, negatively charged N-ZBD exhibits favorable intestinal permeability characteristics. The absorption mechanisms of nanoparticles by intestinal cells primarily involve phagocytosis, clathrin-mediated endocytosis, and macropinocytosis. Research indicates that clathrin mediates the endocytosis of nanoparticles approximately 100 nm in size. This process can circumvent lysosomal degradation, allowing nanoparticles to enter the bloodstream after absorption by intestinal epithelial tissues [7].

Moreover, intestinal epithelial cells contain numerous efflux transporters, such as P-gp protein, which considerably hinder drug absorption. Research has demonstrated that natural nanoparticles from Coptis chinensis extract can encapsulate berberine, facilitating its absorption by Caco-2 cells via indomethacin-sensitive endocytosis. Additionally, these nanoparticles can reduce berberine efflux from intestinal epithelial cells via the P-gp efflux transporter [29]. Both berberine and mangiferin in ZBD are substrates of P-gp [2, 29], indicating that nanoparticles may enhance their absorption in the intestine by inhibiting P-gp expression. Additionally, only dissolved drugs can be absorbed by the intestine, making the solubilization of poorly soluble drugs before reaching the primary absorption site crucial for effective drug therapy. Both mangiferin and berberine are classified as BCS class IV drugs, characterized by poor solubility. N-ZBD significantly enhances the solubility of these compounds, which likely contributes to the notable increase in their bioavailability. The intestinal epithelial layer serves as the primary absorption site for drugs in the small intestine, with two main pathways to cross this barrier: paracellular and transcellular. Tight junctions (TJs) in intestinal epithelial cells constitute a multi-protein complex that forms a relatively stable barrier with low permeability. As a result, hydrophobic drugs find it challenging to cross the intestinal epithelial barrier. These TJs also restrict the paracellular transport of macromolecules, including nanoparticles [29]. Studies indicate that nanoparticles in Coptidis Decoction can regulate the TJs of intestinal epithelial cells, enhancing berberine absorption. Experiments utilizing the Caco-2 cell monolayer model showed that nanoparticles facilitate extracellular transport of berberine by modulating TJs and enhancing intracellular transport through active transport and endocytosis [27]. This suggests that the nanoparticles in ZBD promote drug absorption by opening TJs.

In recent years, nanoparticles in decoctions have garnered significant attention. However, current research primarily centers on isolating nanoparticles and their formation mechanisms. This study revealed that N-ZBD and ZBD exhibit comparable anti-T2DM effects. Additionally, we found that N-ZBD enhances the oral bioavailability of active substances in ZBD for treating type 2 diabetes. Given the prevalence of nanoparticles within herbal decoctions, this study will encourage further studies on nanoparticles in decoctions to better understand their medicinal value.

## Conclusion

This research focused on investigating the anti-T2DM effects of N-ZBD and determining whether it enhances the bioavailability of the primary active compounds in ZBD. The results showed that ZBD significantly mitigated weight loss and reduced FBG, TC, TG, and T-LDL levels in T2DM rats. Among the three phases of ZBD, N-ZBD demonstrated the most effective therapeutic effect, comparable to that of the decoction, indicating that ZBD primarily exerts its anti-T2DM efficacy through N-ZBD. Furthermore, N-ZBD enhances the absorption of active ingredients in the jejunum and ileum compared to free drugs. A UPLC-MS/MS method was developed to measure berberine, mangiferin, neomangiferin, timosaponin BII, phellodendrine, and jatrorrhizine levels in rat plasma following ZBD administration. This method effectively facilitated the comparison of absorption differences among ZBD, N-ZBD, and free drugs. The results indicate that N-ZBD exhibited a pharmacokinetic profile similar to that of ZBD, suggesting a significant enhancement in the oral bioavailability of the active substances. In conclusion, our methods and results may encourage further research on the interactions between active phytochemicals and nanoparticles and their pharmacokinetics in TCM decoctions.

## Supplementary Information

Additional file 1.

## Data Availability

No datasets were generated or analysed during the current study.

## Abbreviations

AUC_0-t_: Area under the curve from zero to time t
AUC_0-∞_: Area under the curve from zero to infinity
*C*_max_: Peak plasma concentration
CLz/F: Clearance adjusted for bioavailability
ELSD: Evaporative Light-Scattering Detector
FBG: Fasting blood glucose
FINS: Fasting serum insulin
IS: Internal standard
*K*_*a*_: The absorption rate constant
LDL: Low Density Lipoprotein
LOQ: Limit of quantification
MRM: Multiple reaction monitoring
MRT_0-t_: Mean residence time from zero to time t
MET: Metformin
N-ZBD: The nanoparticles in ZBD
*P*_*app*_: The apparent permeability coefficient
PDI: Polydispersity index
QC: Quality control
S-ZBD: The sediment phase
TCM: Traditional Chinese medicines
T2DM: Type 2 diabetes mellitus
T-ZBD: The true solution phase
*T*_1/2_: Terminal half-life
*T*_max_: Time to reach peak concentration
TC: Total Cholesterol
TG: Triglyceride
UPLC: Ultra Performance Liquid Chromatography
ZBD: Zhimu and Huangbai decoction
