# Supplementary material for: Zhimu-Huangbai codecoction for the treatment of type II diabetes mellitus through its self-assembling nanoparticles
Source: Chin Med. 2026 Jan 7;21:13. doi: 10.1186/s13020-025-01290-z (PMC12777127; doi:10.1186/s13020-025-01290-z)
Supplement: Supplementary file 1 — Additional file 1. [file 13020_2025_1290_MOESM1_ESM.docx]

Supporting Information

# Zhimu-Huangbai codecoction for the treatment of type II diabetes mellitus through its self-assembling nanoparticles

Wenlong Nie^1,#^, Meifang Jiang^1,2,#^, Yin Li ^5^, Jinshuai Lan^1^, Zhe Li^1^, Zhijun Bi^1^, Donghao Gu^1^, Minquan Zhang^1^, Yue Ding^1,3,4,^*, Tong Zhang^1,3,^*

1 School of Pharmacy, Shanghai University of Traditional Chinese Medicine, Shanghai 201203, China;

2 SPH XingLing Sci&Tech.Pharmaceutical Co.,Ltd., Shanghai, 201707, China.

3 State Key Laboratory of Integration and Innovation of Classic Formula and Modern Chinese Medicine, Shanghai University of Traditional Chinese Medicine, Shanghai 201203, China;

4 National Innovation Platform for medical industry-education integration, Shanghai University of Traditional Chinese Medicine, Shanghai 201203, China；

5 Hebei Open University, Shijiazhuang, Hebei 050080, China.

^#^These authors have contributed equally to this work

*Correspondence:

E-mail address: dingyue-2001@hotmail.com (Yue Ding); zhangtongshutcm@hotmail.com (Tong Zhang).

**Table S1.** Effect of high-speed centrifugation speed on particle size, PDI, and zeta potential of NPs (n=3)

| High-speed centrifugation speed | Size (nm) | PDI | Zeta potential (mV) |
| --- | --- | --- | --- |
| 7000 r/min | 553.1±8.5 | 0.62±0.02 | -13.40±0.46 |
| 10000 r/min | 502.2±14.2 | 0.58±0.01 | -13.63±0.51 |
| 13000 r/min | 367.4±4.8 | 0.55±0.08 | -13.03±0.76 |


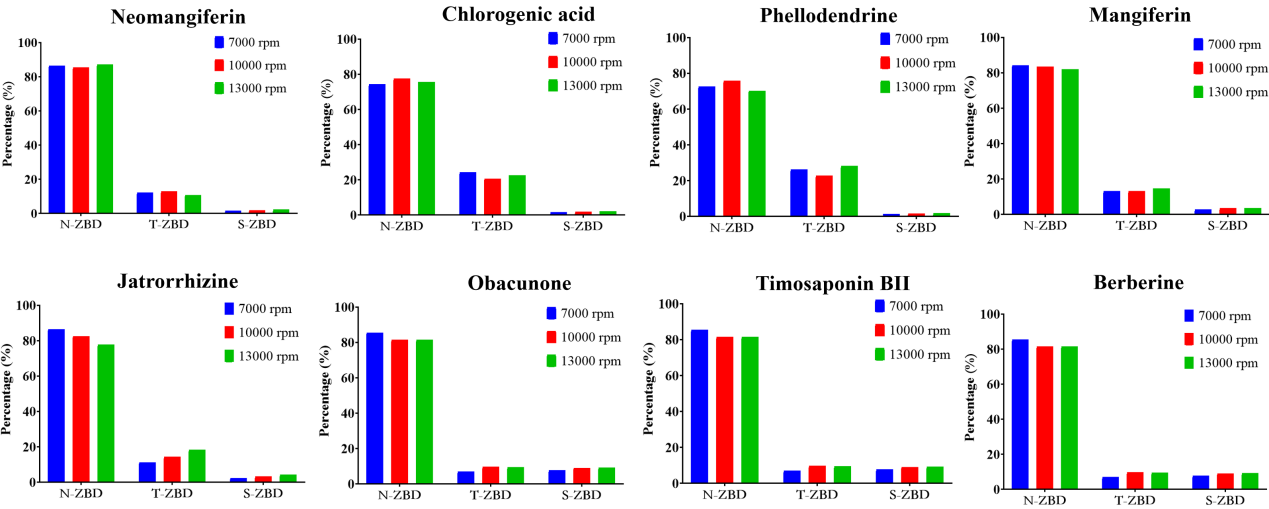


**Fig. S1**. Effect of high-speed centrifugation speed on the content of each component in N-ZBD

**Table S2**. Effect of dialysis membrane molecular weight cut-off on particle size, PDI, and zeta potential of N-ZBD (n=3)

| Dialysis membrane molecular weight cut-off | Size (nm) | PDI | Zeta potential (mV) |
| --- | --- | --- | --- |
| 500 Da | 334.4±1.0 | 0.56±0.06 | -13.27±0.17 |
| 3500 Da | 325.9±5.0 | 0.54±0.03 | -13.46±0.15 |
| 7000 Da | 346.2±9.3 | 0.63±0.05 | -13.58±0.05 |


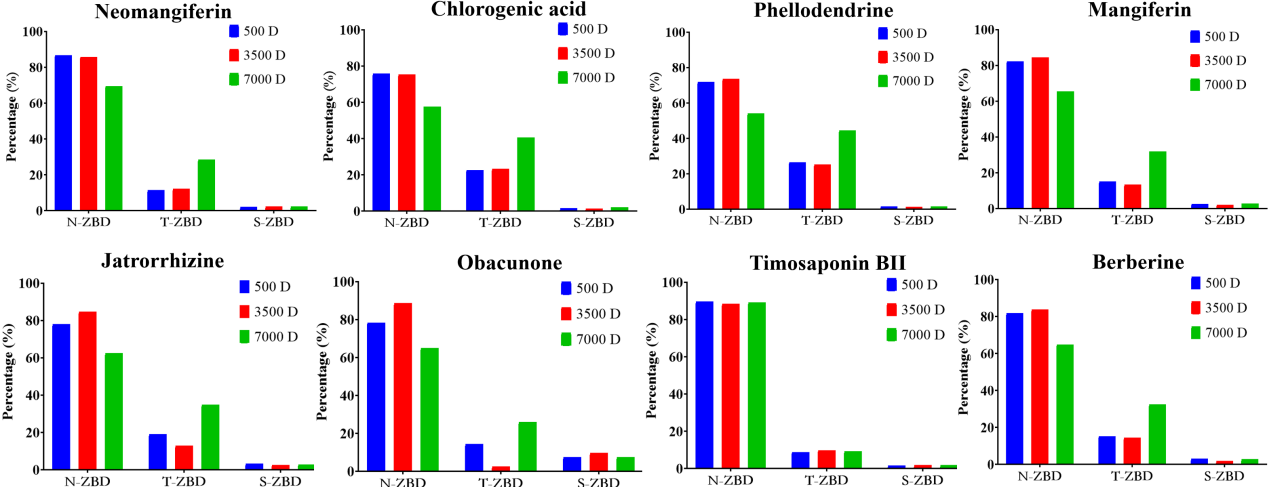


**Fig. S2.** Effect of dialysis membrane molecular weight cut-off on the content of each component in N-ZBD

**Table S3.** Effect of dialysis-centrifugation cycles on particle size, PDI, and zeta potential of NPs（n=3）

| Dialysis-centrifugation cycles | Size (nm) | PDI | Zeta potential (mV) |
| --- | --- | --- | --- |
| 1 | 367.4±4.8 | 0.55±0.08 | -13.03±0.76 |
| 2 | 234.4±1.0 | 0.56±0.06 | -12.97±1.46 |
| 3 | 251.2±5.1 | 0.61±0.01 | -13.37±0.30 |


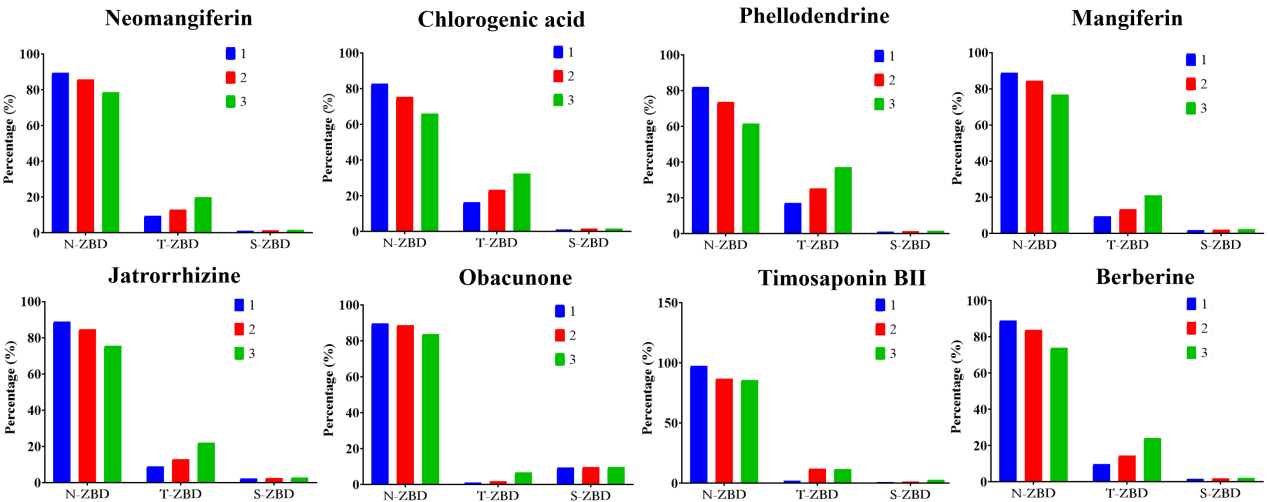


**Fig. S3.** Effect of dialysis-centrifugation cycles on the content of each component in N-ZBD

**
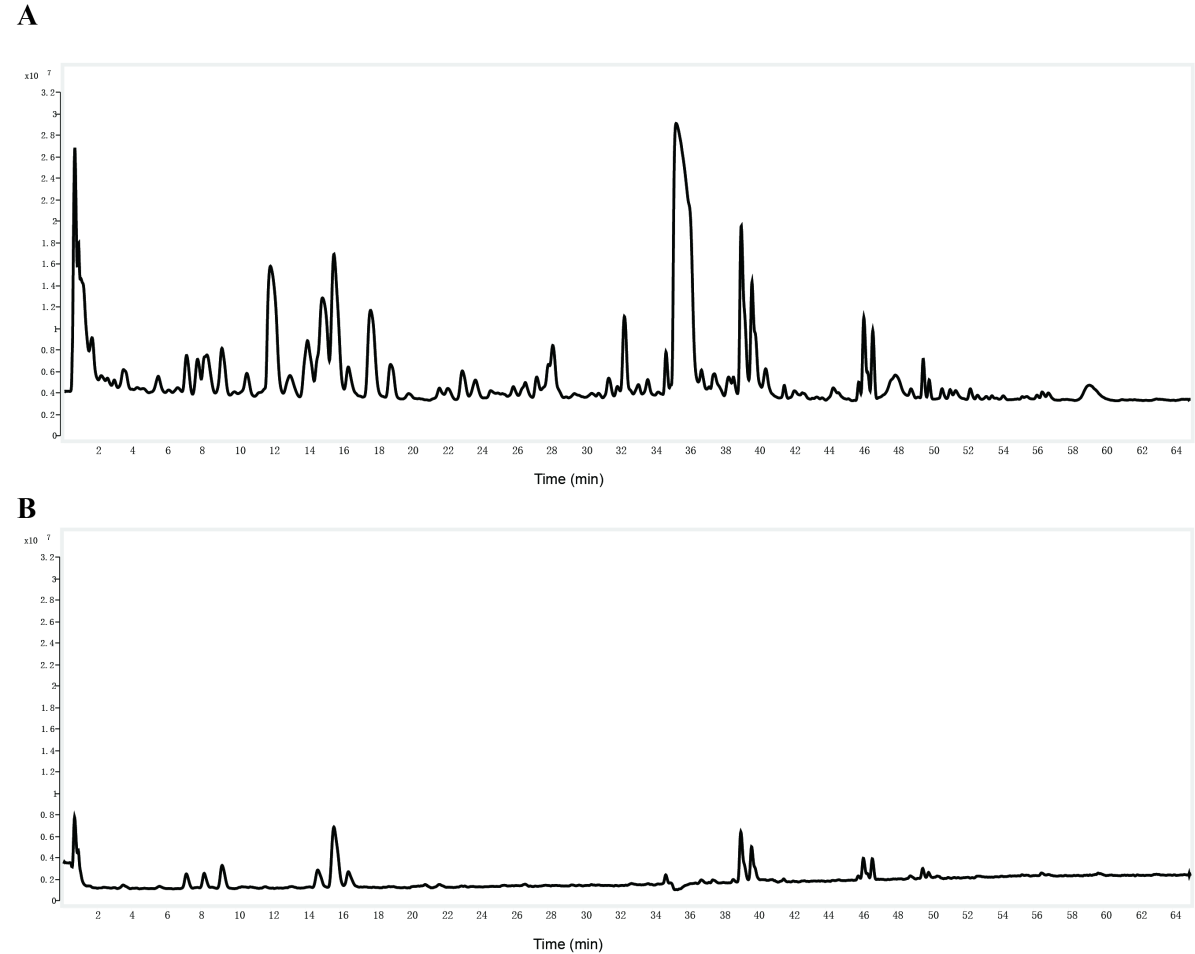
**

**Fig. S4.** The total ion chromatogram (TIC) of N-ZBD. (A) Positive TIC chromograph; (B) Negative TIC chromograph.

**Table S4**. Identification of N-ZBD and mass informations.

| No. | Identity | Formula | t_R_ (min) | Ion mode | Cal *m/z* | *m/z* | Error  (ppm) | MSMS |
| --- | --- | --- | --- | --- | --- | --- | --- | --- |
| 1 * | Chlorogenic acid | C_16_H_18_O_9_ | 7.068 | [M-H]^-^ | 353.0878 | 353.0876 | -0.58 | 191.0559, 127.0396, 85.0294 |
| 2 | 3,4-dihydro-1-[(4-hydroxyphenyl)methyl]-7-methoxy-2-methyl-6-isoquinolinol | C_18_H_19_NO_3_ | 7.758 | [M+H]^+^ | 298.1438 | 298.1438 | 0.44 | 177.0774, 190.0864, 254.1174, 283.1194 |
| 3 | Magnocurarine | C_19_ H_24_NO_3_ | 8.433 | [M]^+^ | 314.1751 | 314.1749 | -0.54 | 107.0491, 237.0902, 269.1157 |
| 4 | Aporphine alkaloid | C_18_H_19_NO_4_ | 9.111 | [M+H]^+^ | 314.1387 | 314.1382 | -1.54 | 151.0749, 298.1061 |
| 5 * | Neomangiferin | C_25_H_28_O_16_ | 9.269 | [M-H]^-^ | 583.1350 | 583.1292 | 0.15 | 301.0346, 331.0454, 463.0869; 493.0924 |
| 6 | Tetrahydroprotoberberine alkaloid | C_20_H_24_NO_4_ | 10.779 | [M]^+^ | 342.1700 | 342.1698 | -0.54 | 192.1018; 279.0995 |
| 7 * | Phellodendrine | C_20_H_24_NO_4_ | 11.909 | [M]^+^ | 342.1700 | 342.1692 | -2.29 | 177.0783; 192.1034 |
| 8 | 4-[(6,7-dimethoxy-2,2-dimethyl-3,4-dihydro-1H-isoquinolin-2-ium-1-yl)methyl]phenol | C_20_H_26_NO_3_ | 13.067 | [M]^+^ | 328.1907 | 328.1900 | -2.19 | 58.0652; 107.0483 |
| 9 | Tembetarine | C_20_ H_26_NO_4_ | 13.789 | [M]^+^ | 344.1856 | 344.1857 | 0.19 | 137.0595; 175.0751 |
| 10 | N-methylhigenamine-7-O-glucopyranoside | C_23_H_29_NO_8_ | 14.072 | [M+H]^+^ | 448.1966 | 448.1969 | 0.68 | 107.0492; 178.0856; 255.1008; 286.1436 |
| 11 | Benzylisoquinoline alkaloid | C_18_H_17_NO_4_ | 14.622 | [M+H]^+^ | 312.1230 | 312.1255 | -0.11 | 177.0788; 268.0959; 297.0986 |
| 12 | Magnoflorine | C_20_H_24_NO_4_ | 14.659 | [M]^+^ | 342.1700 | 342.1695 | -2.29 | 237.0907; 265.0852; 282.0888; 297.1111 |
| 13 * | Mangiferin | C_19_H_18_O_11_ | 14.707 | [M+H]^+^ | 423.0922 | 423.0925 | 0.74 | 405.0809; 303.0495; 387.0705 |
| 14 | Isomangiferin | C_19_H_18_O_11_ | 15.114 | [M+H]^+^ | 423.0922 | 423.0917 | -1.15 | 405.0801; 303.0499 |
| 15 | 2,11-dimethoxy-6,6-dimethyl-5,6,6a, 7-tetrahydro-4H-dibenzo[de, g]quinolin-6-ium-1,10-diol | C_20_H_24_NO_4_ | 15.404 | [M]^+^ | 342.1700 | 342.1699 | -0.25 | 192.1016; 237.0894; 265.0852; 282.0879; 297.1114 |
| 16 | 3-O-feruloylquinic acid | C_17_H_20_O_9_ | 16.320 | [M+H]^+^ | 369.1180 | 369.1179 | -0.3 | 117.0340; 145.0275; 177.0552 |
| 17 | 3-O-feruloylquinic acid | C_17_H_20_O_9_ | 15.669 | [M+H]^+^ | 369.1180 | 369.1188 | 2.14 | 117.0335; 145.0285; 177.0548 |
| 18 | lotusine | C_19_ H_24_NO_3_ | 18.234 | [M+H]^+^ | 314.1751 | 314.1764 | 2.49 | 107.0494; 269.1167; 58.0655; 237.0904 |
| 19 | (1S)-1,2,3,4-tetrahydro-7-hydroxy-1-[(4-hydroxybenzyl)methyl]-2,2-dimethyl-8-O-isoquinolinyl-[3-hydroxy-3-methylglutaryl]-β-D-glucopyranoside | C_42_H_29_N_3_O | 18.950 | [M+H]^+^ | 592.2383 | 592.2392 | 1.45 | 255.1014; 286.1435; 530.2380 |
| 20 | N-methyltetrahydrocolumbamine or N-methyltetrahydrojatrorrhizine | C_21_H_26_NO_4_ | 22.279 | [M]^+^ | 356.1856 | 356.1851 | -1.5 | 177.0775; 192.1017 |
| 21 | Menisperine | C_21_H_26_NO_4_ | 23.075 | [M]^+^ | 356.1856 | 356.1853 | -0.94 | 279.1011; 296.1031; 311.1269 |
| 22 | 6-hydroxy-7-methoxy-1-(4-methoxybenzyl)-2,2-dimethyl-1,2,3,4-tetrahydroisoquinolin-2-ium | C_20_H_26_NO_3_ | 23.397 | [M]^+^ | 328.1907 | 328.1902 | -2.19 | 58.0652; 121.0645; 175.0750; 283.1311 |
| 23 | 2,9-dihydroxy-3,10-dimethoxy-5,6-dihydroisoquinolino[3,2-a]isoquinolin-7-ium | C_19_H_18_NO_4_ | 28.076 | [M]^+^ | 324.1230 | 324.1231 | 0.2 | 280.0961; 294.0755; 308.0913 |
| 24 | Oxyberberine isomer | C_20_H_17_NO_5_ | 28.214 | [M+H]^+^ | 352.1179 | 352.1182 | 0.71 | 294.0740; 308.0910; 322.0705; 336.0858 |
| 25 | Columbamine | C_20_H_20_NO_4_ | 32.057 | [M]^+^ | 338.1387 | 338.1387 | 0.05 | 280.0954; 294.1113; 308.0802; 323.1124 |
| 26 | Thalifendine | C_19_H_16_NO_4_ | 31.592 | [M]^+^ | 322.1074 | 322.1072 | -0.57 | 279.0881; 307.0835 |
| 27 | Columbamine | C_20_H_20_NO_4_ | 31.930 | [M]^+^ | 338.1387 | 338.1391 | -0.39 | 280.0943; 294.1118; 308.0914; 322.1066 |
| 28 * | Jatrorrhizine | C_20_H_20_NO_4_ | 32.437 | [M]^+^ | 338.1387 | 338.1391 | -0.39 | 279.0882; 280.0954; 294.1118; 306.1118; 308.0906; 322.1068 |
| 29 | Groenlandicine | C_19_H_16_NO_4_ | 33.752 | [M]^+^ | 322.1074 | 322.1070 | -1.19 | 209.0116; 307.0834 |
| 30 * | Berberine | C_20_H_18_NO_4_ | 35.647 | [M]^+^ | 336.1230 | 336.1236 | 1.68 | 278.0811; 292.0975; 306.0763; 320.0930 |
| 31 * | Timosaponin BII | C_45_H_76_O_19_ | 39.127 | [M-H]^-^ | 919.4908 | 919.4891 | 0 | 433.3323; 595.3852; 757.4357 |
| 32 | Timosaponin C | C_45_H_74_O_18_ | 39.295 | [M+H]^+^ | 903.4948 | 903.4938 | -1.1 | 579.3876; 741.4400 |
| 33 | Timosaponin B | C_45_H_74_O_18_ | 39.778 | [M+H]^+^ | 903.4948 | 903.4948 | 0.01 | 579.3876; 741.4399 |
| 34 | Timosaponin A2 | C_39_H_64_O_14_ | 41.589 | [M+H]^+^ | 757.4369 | 757.4352 | -2.22 | 593.3587 |
| 35 * | Limonin | C_26_H_30_O_8_ | 48.995 | [M+H]^+^ | 471.2013 | 471.2010 | -0.73 | 425.1877; 161.0597; 95.0123 |
| 36 | Timosaponin AIII | C_39_H_64_O_13_ | 49.389 | [M+H]^+^ | 741.4420 | 741.4416 | -0.5 | 577.3735 |
| 37 | 22β-timosaponin AIII | C_39_H_64_O_13_ | 49.664 | [M+H]^+^ | 741.4420 | 741.4400 | -2.66 | 577.3715 |
| 38 * | Obacunone | C_26_H_30_O_7_ | 54.892 | [M+H]^+^ | 455.2064 | 455.2064 | -0.07 | 391.1890; 409.1978; 437.1973 |

* The compound was confirmed by comparison with the reference standard.


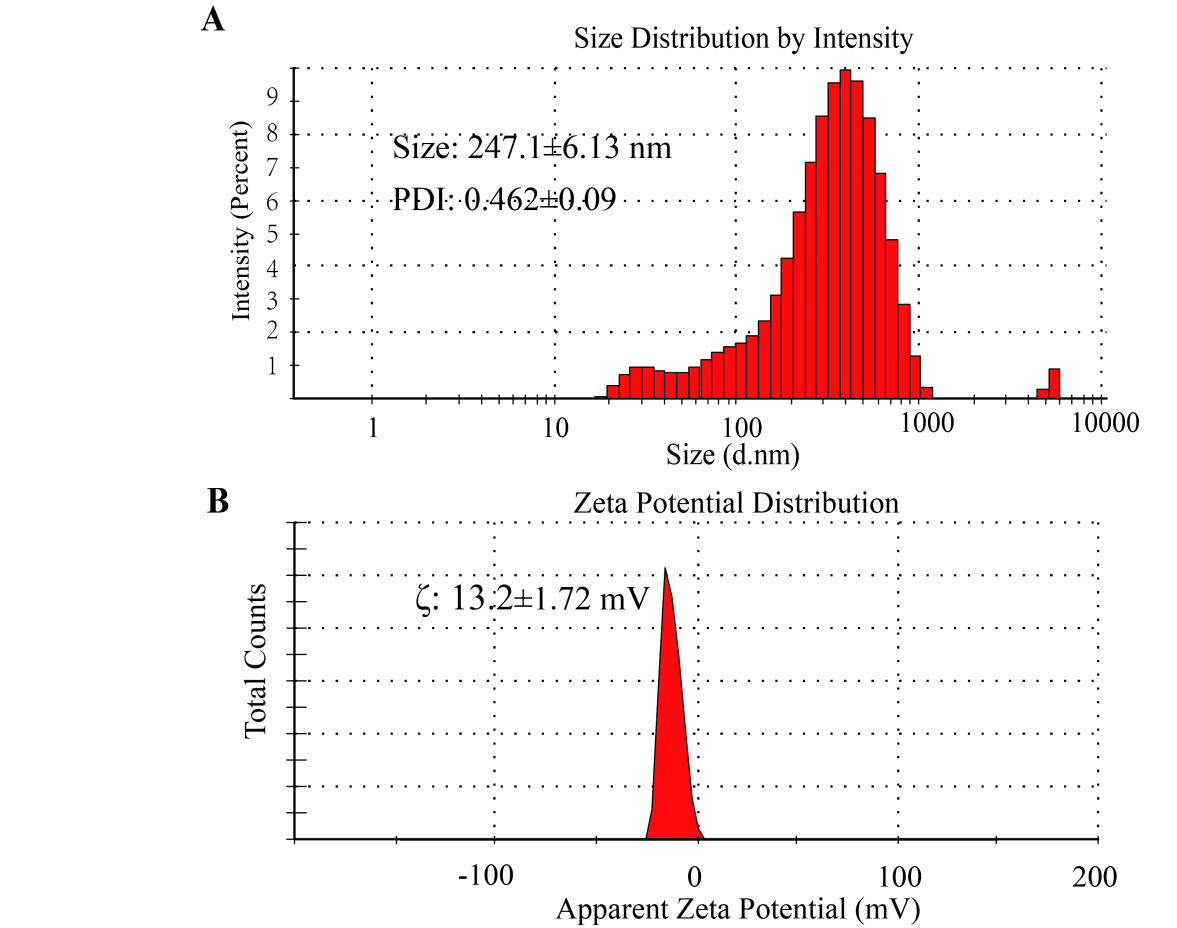


**Fig. S5.** (A) Particle size distribution of N-ZBD after concentration and subsequent dilution. (B) The zeta potential of N-ZBD after concentration and subsequent dilution.
